# Comparing maternal genetic variation across two millennia reveals the demographic history of an ancient human population in southwest Turkey

**DOI:** 10.1098/rsos.150250

**Published:** 2016-02-17

**Authors:** Claudio Ottoni, Rita Rasteiro, Rinse Willet, Johan Claeys, Peter Talloen, Katrien Van de Vijver, Lounès Chikhi, Jeroen Poblome, Ronny Decorte

**Affiliations:** 1 Center for Archaeological Sciences, University of Leuven, Leuven, Belgium; 2 Department of Imaging & Pathology, University of Leuven, Leuven, Belgium; 3 Sagalassos Archaeological Research Project, University of Leuven, Leuven, Belgium; 4 Laboratory of Forensic Genetics and Molecular Archaeology, UZ Leuven, Belgium; 5 Department of Genetics, School of History, University of Leicester, Leicester, UK; 6 Depatment of Humanities, Institute of History, Leiden University, Leiden, The Netherlands; 7 CNRS, Université Paul Sabatier, ENFA; UMR5174 EDB (Laboratoire Évolution & Diversité Biologique), Toulouse, France; 8 Université Toulouse 3 Paul Sabatier, CNRS; UMR5174 EDB, Toulouse, France; 9 Instituto Gulbenkian de Ciência, Oeiras, Portugal

**Keywords:** ancient DNA, approximate Bayesian computation, Turkey, Roman, Byzantine

## Abstract

More than two decades of archaeological research at the site of Sagalassos, in southwest Turkey, resulted in the study of the former urban settlement in all its features. Originally settled in late Classical/early Hellenistic times, possibly from the later fifth century BCE onwards, the city of Sagalassos and its surrounding territory saw empires come and go. The Plague of Justinian in the sixth century CE, which is considered to have caused the death of up to a third of the population in Anatolia, and an earthquake in the seventh century CE, which is attested to have devastated many monuments in the city, may have severely affected the contemporary Sagalassos community. Human occupation continued, however, and Byzantine Sagalassos was eventually abandoned around 1200 CE. In order to investigate whether these historical events resulted in demographic changes across time, we compared the mitochondrial DNA variation of two population samples from Sagalassos (Roman and Middle Byzantine) and a modern sample from the nearby town of Ağlasun. Our analyses revealed no genetic discontinuity across two millennia in the region and Bayesian coalescence-based simulations indicated that a major population decline in the area coincided with the final abandonment of Sagalassos, rather than with the Plague of Justinian or the mentioned earthquake.

## 1. Background

In the last three decades, palaeogenetics, the study of ancient DNA (aDNA), has proven invaluable to disentangle the biological and cultural processes in human (pre)history. The time-depth provided by the analysis of genetic variation in past populations is key to circumventing some of the limitations of interpreting present-day genetic patterns alone. In particular, the study of the maternally inherited mitochondrial DNA (mtDNA) has contributed to reveal past demographic scenarios previously undetected in ancient human communities.

Within the Eurasian continent, most aDNA studies on human populations have addressed various levels of temporal and geographical resolution, from wide-scale prehistoric population dynamics [1,2] to smaller-scale demographic events in more recent historical times [3,4].

Across multiple temporal layers of the history of our species, the importance of the Anatolian peninsula as a bridge between continents and cultures is unquestionable. The remains of the ancient city of sagalassos are situated in southwest Turkey, at an altitude of 1450–1600 m above sea level in the Taurus mountain range. Located 7 km north of the town of Ağlasun, in the Turkish province of Burdur (electronic supplementary material, figure S1), Sagalassos played a key role in the past of the wider region then known as Pisidia, as almost 30 years of archaeological research have revealed [5].

The first indications of human presence in the Ağlasun Valley can be placed at approximately 10 000 BCE in Epi-Palaeolithic times. The study region, which forms part of the Turkish Lake District, contains a range of well-studied Late Neolithic and Bronze Age sites [6]. The so-called Beysehir occupation phase (BO-phase) introduced a period of increased anthropogenic activity from around 1000 BCE, although the start date can vary quite significantly between specific areas. A hierarchical settlement landscape emerged during the Iron Age in the wider region. Finally, in late Classical/early Hellenistic times (possibly from the later fifth century BCE onwards), Sagalassos and Düzen Tepe—an adjacent settlement that was inhabited into the second century BCE—originated together with a group of small farms in the surrounding valley. From its origins up to its abandonment in the early thirteenth century CE, Sagalassos formed part of different empires administering Pisidia, namely that of the Achaemenids, the Hellenistic Successor Kings, the Roman and Byzantine Empires, and possibly their regional Seljuk sequel [7].

During the *Pax Romana*, a period of peace and reduced military engagement spanning more than two centuries from 25 BCE up to 180 CE, Sagalassos experienced one of its most flourishing periods. This is testified by an intense pottery production, large-scale exchange [8] and expansive urbanization into the early third century (electronic supplementary material, text). Sagalassos remained a densely occupied urban centre at least until the mid-sixth century. Afterwards, the urban framework changed, including the abandonment of some quarters in the town.

Two main factors are considered to have contributed to the traditional scenario of late antique (demographic) decline and the overall restructuring of Sagalassos’ community. One factor is the first recorded outbreak of plague—the Plague of Justinian—which between 541 and 543 CE spread into Europe through the Mediterranean Basin [9], causing the death of up to a third of the population in Anatolia and the Eastern Mediterranean [10]. Pisidia may have suffered the same fate, even though no clear evidence of the spread of the plague up to Sagalassos and its wider region is so far present.

The second factor is seismic activity in the area. A severe earthquake destroyed several monuments in the urban centre in the course of the first half of the seventh century CE [11]. The community of Sagalassos lived on, albeit in different conditions, possibly resembling the larger villages that the Roman emperor Justinian associated with the region of Pisidia [12].

Whereas the demographic trajectories of the following centuries remain quite elusive, at least based on the current archaeological and historical evidence, the final abandonment of Sagalassos can be dated to the early thirteenth century CE, when a fortified settlement or *kastron* was destroyed, and the local population is considered to have shifted towards Ağlasun. By that time, Ağlasun was occupied by the Seljuk Turks [13] and is assumed to have been continuously inhabited up to the present time.

To better elucidate the demographic changes across time that affected the people living in Sagalassos and its territory, we have analysed the mtDNA variation in 24 individuals dated to Roman and Early Byzantine times, ranging from the early second up to the sixth century CE. The mtDNA sequences obtained were compared with a Middle Byzantine sample from Sagalassos previously studied [14] and with modern mtDNA sequences from the town of Ağlasun [15] (electronic supplementary material, table S2), which nowadays accounts for about 4100 inhabitants (http://www.tursaga.com/). Finally, we applied Bayesian coalescence-based approaches to identify the most plausible demographic scenarios supported by available DNA, historical and archaeological evidence.

## 2. Methods

### 2.1 Genetic analyses

Bone and teeth samples of 44 individuals dated to late Hellenistic (late first century BCE), Roman Imperial (first–third centuries CE), Late Roman (fourth–fifth centuries CE) and Early Byzantine times (sixth–seventh centuries CE), as determined by direct accelerator mass spectrometry radiocarbon dating, stratigraphic associations and contextual archaeological evidence, were available for aDNA analyses (electronic supplementary material, table S1).

Analyses were carried out in dedicated aDNA facilities of the Forensic Genetics and Molecular Archaeology Department of the University of Leuven (Belgium). The two hypervariable segments (HVS-I and HVS-II) of the mtDNA and eight diagnostic SNPs of the mtDNA coding region were amplified and sequenced in multiple independent experiments as described elsewhere [14]. Further details regarding methods, precautions taken to minimize contamination, and the criteria adopted to authenticate the aDNA sequences are described in the electronic supplementary material, text.

### 2.2 Data analyses

To unravel the genetic affinities of two ancient population samples from Sagalassos (Roman and Middle Byzantine) and the modern sample from Ağlasun to present-day populations, we generated an in-house database with more than 11 000 concatenated HVS-1 and HVS-2 mtDNA sequences of contemporary populations from the literature (electronic supplementary material, table S7). We used Arlequin v. 3.5 [16] to compute pairwise *F*_ST_ genetic distances. The Slatkin’s linearized *F*_ST_ values were represented in a two-dimensional multidimensional scaling plot with Statistica v. 8 (Statsoft) and for Sagalassos and Ağlasun they were plotted on a geographical map with Surfer v. 6 (Golden Software) using the Kriging method. The relative frequencies of haplotypes from Sagalassos and Ağlasun (excluded CRS haplotypes) shared with the modern populations were estimated with Arlequin and displayed on a geographical map with Surfer. A median-joining network [17] of concatenated HVS1–HVS2 haplotypes from Sagalassos and Ağlasun was created with Network v. 4.6 (www.fluxus-engineering.com).

To include a larger number of populations and ancient samples from the literature, a second database of more than 17 000 HVS-1 sequences was used for *F*_ST_ genetic distances analyses (electronic supplementary material, table S8).

Haplogroup frequencies from more than 180 ancient and modern populations were used for a principal component analysis (PCA) and represented in a two-dimensional plot with Statistica (electronic supplementary material, table S9). Detailed information about the statistical methods and the databases is given in the electronic supplementary material.

### 2.3 Simulation analyses

**Figure 1.**
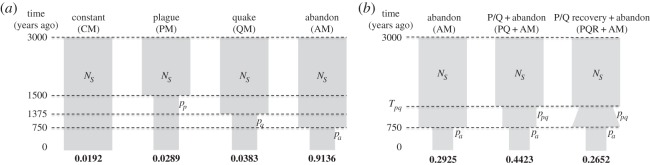
Demographic scenarios used in the ABC analysis and their posterior probabilities. Two sets of different demographic scenarios were tested using ancient and modern mtDNA. In panel (*a*), three simple scenarios of population size change were compared to study the impact of a single past population contraction following the Plague of Justinian (PM), the seventh century earthquake (QM) and the abandonment (AM) of the city of Sagalassos. These scenarios assume a single constant female effective population size *N*_*S*_, prior to the contraction, sampled from an ancestral female population of constant size *N*_*anc*_, corresponding to the initial settlers of the region. A scenario of no size change (CM) since the BO-phase in the region until the present in Ağlasun was also tested as a null hypothesis. The posterior probabilities identified the AM scenario as the best model. In panel (*b*), scenarios with a second contraction added to the AM scenario were studied. This population size contraction corresponds to a size reduction during the plague and/or earthquake at time *T*_*pq*_ and was followed by either a period of recovery (PQR+AM) or constant population size (PQ+AM) until the abandonment of the city of Sagalassos. The parameter *p*_*i*_ (*p*_*p*_, *p*_*q*_ and *p*_*a*_ for plague, quake and abandonment of the city, respectively) represents the proportion used to calculate the effective population size after the contraction (*N*_*C*_) at time *i*, such that *N*_*C*_=*p*_*i*_×*N*_*S*_ for scenarios AM, PM and QM. In the more complex scenarios, *N*_*C*_ after the abandonment of the city is *p*_*pq*_×*p*_*a*_×*N*_*S*_ (*p*_*pq*_ being the contraction proportion at time *T*_*pq*_) and *p*_*a*_×*N*_*S*_ for PQ+AM and PQR+AM, respectively. Both in (*a*) and (*b*), the posterior probabilities under each model are represented, calculated using the multinomial logistic regression (MLR) method of Beaumont [18], under an ABC framework.

In order to identify the major events that took place during the history of Sagalassos, we first built demographic models (figure 1) involving either no change in population size or a population size change during one of the known catastrophic events that took place (plague, earthquake, abandonment). To compare the different models and identify those that best explain the data, we used an approximate Bayesian computation (ABC) framework [18,19] and implemented it together with the Bayesian serial Simcoal software [20,21], a program that simulates ancient and modern mtDNA data with sample sizes and dates identical to the real data. We also applied two different methods to test for continuity versus discontinuity between the two ancient samples from Sagalassos (Roman and Middle Byzantine) and Ağlasun (electronic supplementary material, text and figure S8).

Further details regarding the ABC validation, continuity tests and additional simulations accounting for potential maternal relationships in the Roman sample are fully described in the electronic supplementary material.

## 3. Results

### 3.1 Mitochondrial DNA genetic pool in Sagalassos across time

Authentic aDNA sequences were obtained for 22 Roman and two Early Byzantine individuals (electronic supplementary material, table S2), which were pooled in one population sample addressed here as Roman. Haplogroup frequencies in Roman and Middle Byzantine Sagalassos and Ağlasun (table 1) show overlapping 95% Bayesian credible intervals except for macro-haplogroup M in Middle Byzantine Sagalassos and Ağlasun.

**Table 1.** Absolute and relative frequencies of mtDNA haplogroups. This table presents the estimated frequencies together with the 95% Bayesian credible intervals (CI), in Roman and Middle Byzantine Sagalassos and in the modern town of Ağlasun.

	Roman Sagalassos		Middle Byzantine Sagalassos^a^		Ağlasun^b^	
	% (N)	CI	% (N)	CI	% (N)	CI
R0/HV/H	17 (4)	7–36	29 (15)	19–43	25 (13)	15–38
I	0	0–14	0	0–7	2 (1)	0–10
N1	0	0–14	12 (6)	6–23	0	0–7
J	0	0–14	12 (6)	6–23	8 (4)	3–18
T	17 (4)	7–36	10 (5)	4–21	17 (9)	9–29
U1	4 (1)	1–20	4 (2)	1–13	0	0–7
U2	0	0–14	0	0–7	6 (3)	2–15
U3	0	0–14	6 (3)	2–16	0	0–7
U4	4 (1)	1–20	0	0–7	0	0–7
U5	4 (1)	1–20	2 (1)	0–10	4 (2)	1–13
U6	0	0–14	4 (2)	1–13	0	0–7
U7	4 (1)	1–20	0	0–7	0	0–7
U8 (incl. K)	29 (7)	15–49	12 (6)	6–23	13 (7)	7–25
W	4 (1)	1–20	8 (4)	3–19	2 (1)	0–10
X	17 (4)	7–36	2 (1)	0–10	0	0–7
M (incl. C)	0	0–14	0	0–7	15 (8)	8–27
?	0	0–14	0	0–7	9 (5)	4–20
Total (N)	24		51		53	

^a^Ottoni *et al*. [14].

^b^Jehaes [15].

The high diversity of mtDNA lineages observed in Sagalassos and Ağlasun is exhibited by the network (electronic supplementary material, figure S2). In several instances, haplotypes of the three samples (haplogroups W, K1, T2, U5a, H) differ by one or two mutational steps.

When compared with haplotypes of the database, Sagalassos shows a lower fraction of unique haplotypes (43% and 41%, respectively, in the Roman and Middle Byzantine samples) than Ağlasun (57%) and overall the haplotypes shared with present-day populations have a West Eurasian distribution (electronic supplementary material, figure S3 and tables S5–S6).

**Figure 2.**
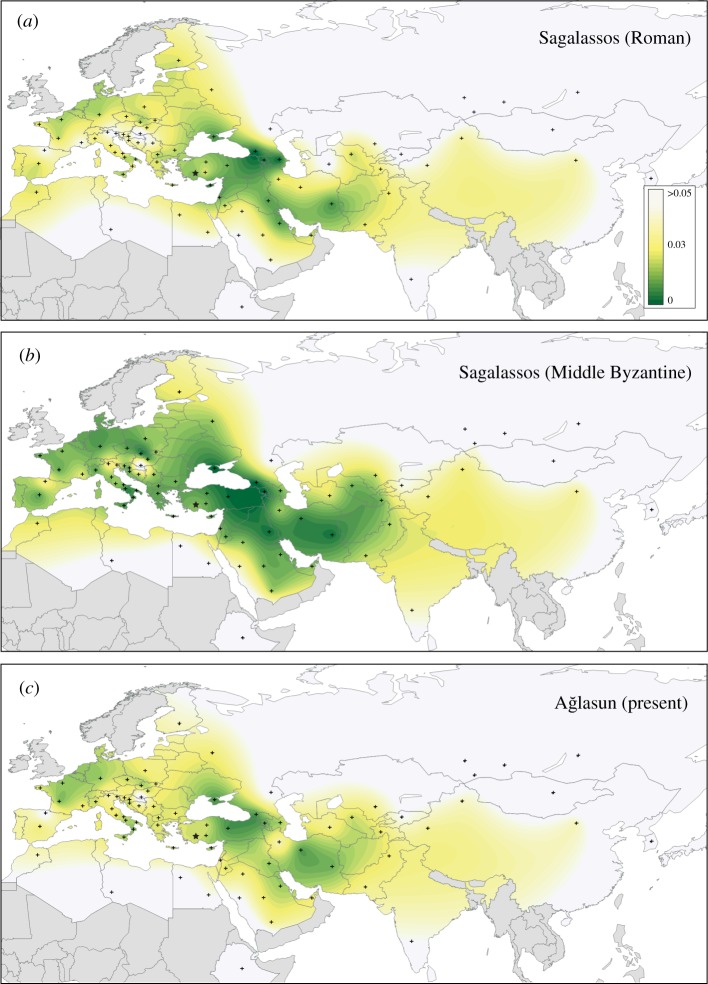
Contour maps of pairwise *F*_ST_ values between each of the three chronological samples of this study, Roman Sagalassos (*a*), Middle Byzantine Sagalassos (*b*), modern Ağlasun (*c*), and 77 modern populations from the database. Genetic distances are represented by a gradient from green (smallest *F*_ST_ values) to white (largest *F*_ST_ values). Approximate location of samples used for the analysis is indicated by a cross. Countries without any samples available are shaded. The location of Sagalassos is indicated by a star.

Figure 2 represents the pairwise genetic distances calculated from the database of HVS-1 and HVS-2 sequences on a geographical map. A typical West Eurasian variation in Sagalassos during Roman times is evident (figure 2*a*) and the *F*_ST_ values indicate in particular higher affinity with populations from the Caucasus, Southwest Asia, the Eastern Mediterranean, Southern Italy and some countries of continental Europe (Germany, Northern France; electronic supplementary material, table S3 and figure S4a). Northern Asian populations exhibited the largest values. A similar pattern of genetic affinities is observed in Middle Byzantine Sagalassos and in Ağlasun (figure 2*b*,*c*).

A Southwest Asian affinity of Sagalassos and Ağlasun is also detected when contrasting genetic distances with a larger number of populations at the level of HVS-1 variation (electronic supplementary material, table S4 and figures S4b and S6). Ancient Mesolithic, Neolithic and Bronze Age samples from Europe and Asia were included in the analysis and some from Early–Middle Neolithic central Europe [1] showed proximity with Sagalassos.

Concordant with the *F*_ST_ analysis, the haplogroup frequencies PCA (electronic supplementary material, figure S7) reveals affinity between ancient Sagalassos and Southwest Asian populations, and an even closer relationship with samples from Caucasus, Southern Italy (Sicily, Apulia, Campania) and the ancient samples of Early and Middle Neolithic central European farmers and Bronze Age Crete.

### 3.2 Identifying, quantifying and dating population size changes

Figure 1 represents the two sets of demographic scenarios tested together with their posterior probabilities [22]. These scenarios assume continuity between the Sagalassos and Ağlasun samples, and this continuity was tested and not rejected (electronic supplementary material, text and figure S8). In figure 1*a*, the model with the highest support is the AM (91%), where the population contraction corresponding to the abandonment of the city of Sagalassos is very strong (median value 93%, table 2). In figure 1*b*, we further explored this scenario by adding a second contraction at time *T*_*pq*_, corresponding to the plague and/or the earthquake, but the method could not distinguish between the three scenarios. Furthermore, the parameters estimated for the scenarios in figure 1*b* suggest that if there was a population size contraction at time *T*_*pq*_, it was small (table 2) with median values ranging between 10% and 20%. The median values for the contraction corresponding to the abandonment of Sagalassos are very similar (90%) to the value found when there is only one major contraction (the AM scenario). Hence, the data suggest one major demographic event corresponding to the abandonment of the city but it cannot be determined whether the earthquake or the plague also contributed to changes in genetic diversity.

**Table 2.** Demographic parameters for the scenarios described in figure 1*b*. The posterior weighted (*ω*) median, 5% and 95% percentile values are represented for the parameters described in figure 1*b*. The priors were taken from broad uniform distributions, ranging between a minimum and maximum value (*U*: min–max), for each of the parameters. The *T*_*pq*_ parameter represents the time in generations ago (assuming that one generation corresponds to 25 years and that the present is set to 2000 CE) for a possible size contraction in Sagalassos, prior to the abandonment of the city, and it has a range of 15 generations to include the plague outbreak and seventh century earthquake.

			posterior		
scenarios	parameters	prior	*ω*P5%	*ω*median	*ω*P95%
AM	*p*_*a*_	*U*: 0.01–1	0.0438	0.0774	0.2211
	*N*_*S*_	*U*: 1000–40 000	20 227	33 550	39 506
	*N*_*anc*_	*U*: 1000–5000	2504	4062	4916
PQ+AM	*T*_*pq*_	*U*: 50–65	50	53	63
	*p*_*pq*_	*U*: 0.2–1	0.3581	0.7701	0.9777
	*p*_*a*_	*U*: 0.01–1	0.0325	0.1086	0.5703
	*N*_*S*_	*U*: 1000–40 000	15 413	32 032	39 750
	*N*_*anc*_	*U*: 1000–5000	3219	4187	4828
PQR+AM	*T*_*pq*_	*U*: 50–65	51	56	65
	*p*_*pq*_	*U*: 0.2–1	0.4271	0.8939	0.9950
	*p*_*a*_	*U*: 0.01–1	0.0371	0.0806	0.3288
	*N*_*S*_	*U*: 1000–40 000	11 923	32 272	39 820
	*N*_*anc*_	*U*: 1000–5000	2204	4109	4975

## 4. Discussion

### 4.1 The mtDNA pool in the Sagalassos study region across time

The maternal genetic composition of Sagalassos in Roman times shows a typical West Eurasian pool, as previously observed in the Middle Byzantine sample [14]. The multiple analyses carried out at the level of mtDNA genetic variation (*F*_ST_ genetic distances, PCA, shared haplotypes) reveal a clear Southwest Asian affinity, in particular with the Caucasus, Iraq, Iran, the Levant and the general Anatolian region. Genetic distances increase eastward across the Asian continent, and westward in Europe, though a genetic affinity was observed with continental Western Europe (France and Germany) and Southern Italy. Furthermore, the analyses reveal a common genetic thread along Southwest Asia, the Mediterranean basin (e.g. Southern Italian and Greek samples, including Bronze Age Crete) up to ancient central European farming communities, a pattern that may reflect the complex connections that took place in these areas across time since the Neolithic [23,24].

Roman times were certainly dynamic in the whole Mediterranean region. In that period, Sagalassos grew into the major regional centre of Pisidia. The city expanded and grounded its economic success in local agricultural and artisanal production [25]. As a dynamic centre in an empire characterized by mobility, Sagalassos may have attracted people from all over the Mediterranean and the Near East to some degree. Remarkably, the emperor Augustus established colonies of Roman veterans from Italy—with attested origins in the region of Campania—and Southern Spain in the region of Pisidia upon its incorporation into the Empire after 25 BCE [7]. Overall, such a scenario may explain the high diversity and the degree of affinity of Sagalassos with West Eurasian populations across Southwest Asia and the Mediterranean basin.

The mtDNA pool of Sagalassos and Ağlasun remained homogeneous across time, in fact no significant differences in haplogroup frequencies were observed, except for macro-haplogroup M. This is corroborated by the network analysis, which showed close genealogical relationships between most of the mtDNA lineages in the three samples.

In agreement with published data from modern Turkish populations [26], lineages of East Eurasian descent assigned to macro-haplogroup M were found in the modern sample from Ağlasun. This haplogroup is significantly more frequent in Ağlasun (15%) than in Byzantine Sagalassos, where it is absent (the non-significant value of the Roman sample might be most likely due to its low sample size), thus indicating that this East Eurasian component may have been introduced later, either recently or even as early as the Seljuk invasion of Turkey in the eleventh century CE.

Middle Byzantine Sagalassos overall showed lower genetic distances with the modern populations as opposed to the Roman sample and Ağlasun (figure 2). This could be a mere sampling effect, nevertheless the older chronological age of the Roman sample on the one hand, and isolation and drift effects in Ağlasun (as opposed to the high dynamicity of ancient Sagalassos) on the other hand, may explain a similar pattern (electronic supplementary material, text).

### 4.2 Demographic change

Our results support population continuity until the present in the study region of Sagalassos since Roman times, suggesting that, even though migratory events may have taken place across the centuries (e.g. linked to the introduction of Asian lineages), no significant major changes that could not be explained by a relatively simple demographic model occurred in the mtDNA pool of the people inhabiting the area.

There is very scarce direct evidence of human presence in the immediate surroundings of Sagalassos and the Ağlasun Valley before the origins of the human communities in the later fifth century BCE. However, in all demographic scenarios tested we set the BO-phase (approx. 1000 BCE) as the initial date of human occupation of the region in light of the strong increase in cereal cultivation, arboriculture and grazing grounds for livestock, which are indicative of a significant increase of human activities in the area [27].

Our ABC analyses strongly support a scenario in which a population decline in the study region coincided with the abandonment of Sagalassos in the early thirteenth century CE, rather than with the Plague of Justinian or the earthquake in the seventh century CE (figure 1*a*).

We further explored this scenario in more depth by investigating whether the plague and/or the earthquake contributed to a reduction in population size in the region prior to the abandonment of the city. We also included a scenario of slight recovery in population size between the two contractions (figure 1*b*). Our simulations did not make it possible to discriminate with high support any of the three alternative scenarios investigated. However, cumulative posterior probabilities of the models with two contractions (71%) suggest that a decline coincident with the plague and/or the earthquake may have taken place. If it occurred, then it was moderate, with a reduction of 10–20% in effective population size in the general study region. Whether this contraction was due to an increased death rate or to migration out of the region remains to be investigated.

Most likely, either the plague and/or the earthquake in the seventh century CE determined a general loss of social complexity of Sagalassos’ community [12], which would continue to wax and wane into the early thirteenth century. By then, the wider region had seen the arrival of the Seljuk Turks, who are attested to have built a caravanserai and *hamam* in Ağlasun in the 1230s CE [13]. We cannot exclude that at least part of the population of Sagalassos moved upon its abandonment, or even earlier, to Ağlasun. Nevertheless, our simulations indicate that concurrently with the abandonment of Sagalassos, a reduction of at least 90% in the effective population size of the general study region took place, suggesting that local people probably moved to more distant areas in southwest Anatolia.

As a final consideration, after accounting for the effective population size of mtDNA (one-quarter of the autosomal DNA) and the effective size as approximately one-third of the census size, the results of our simulations indicate a wide discrepancy with the census size estimated through archaeological survey—up to 50 000 including the urban and rural area (electronic supplementary material, text)—prior to any possible demographic contraction. These results should be treated with caution, owing to possible underestimations in the methodology of the archaeological survey, and to the limited power of demographic reconstructions based on a single genetic locus. In addition, the effective population sizes estimated from genetic data can be difficult to relate to actual population sizes when populations are structured. In such cases, the estimated effective size will be influenced by the area over which gene flow occurs.

The discrepancy observed might suggest that the mtDNA pool of people in the region was enriched across time by the introduction of novel lineages, thus indicating more complex scenarios and supporting the hypothesis of genetic signatures left by mobility into the area. The models tested are clearly not exhaustive of all possible demographic factors that may have taken place across time in the study region. Modelling the source and extent of gene flow in the study region of Sagalassos was beyond the scope of this study, but may become an interesting focus of future studies when additional aDNA data can be obtained across wider regions of Anatolia and the surrounding regions.

## 5. Conclusion

In this study, we addressed questions regarding the demographic trajectories across the last two millennia of a human population at the very fine scale of one city—Sagalassos, in southwest Turkey—and its surrounding region. By comparing mtDNA variation in three period groups, Roman, Middle Byzantine and modern, and by simulating possible scenarios inferred by historical and archaeological evidence, our data suggest that concurrently with the abandonment of the city in the early thirteenth century CE, the population of the region may have been drastically reduced by almost 90%, most likely owing to migrations towards farther regions in southwest Anatolia. Furthermore, an earlier but milder contraction in population size may have taken place in the sixth–seventh centuries CE, either owing to the Plague of Justinian and/or an earthquake in the region. Whether this hypothetical contraction was due to increased death rate or migration out of the region remains unclear, and—together with the overall demographic scenario described here—needs to be better addressed by incorporating nuclear markers on a large genomic scale.

## Supplementary Material

1 supplementary file containing 9 supplementary figures S1-S9.

## Supplementary Material

1 supplementary dataset containing 9 supplementary tables.

## Supplementary Material

1 supplementary text file with a detailed description of the methods used in the present study.

## Data accessibility

The DNA sequences generated in this study are available in GenBank (accession number KT892706–KT892729).
