# Supplementary material for: Comparing maternal genetic variation across two millennia reveals the demographic history of an ancient human population in southwest Turkey
Source: R Soc Open Sci. 2016 Feb 17;3(2):150250. doi: 10.1098/rsos.150250 (PMC4785964; doi:10.1098/rsos.150250)
Supplement: 1 supplementary file containing 9 supplementary figures S1-S9. [file rsos150250supp1.pdf]

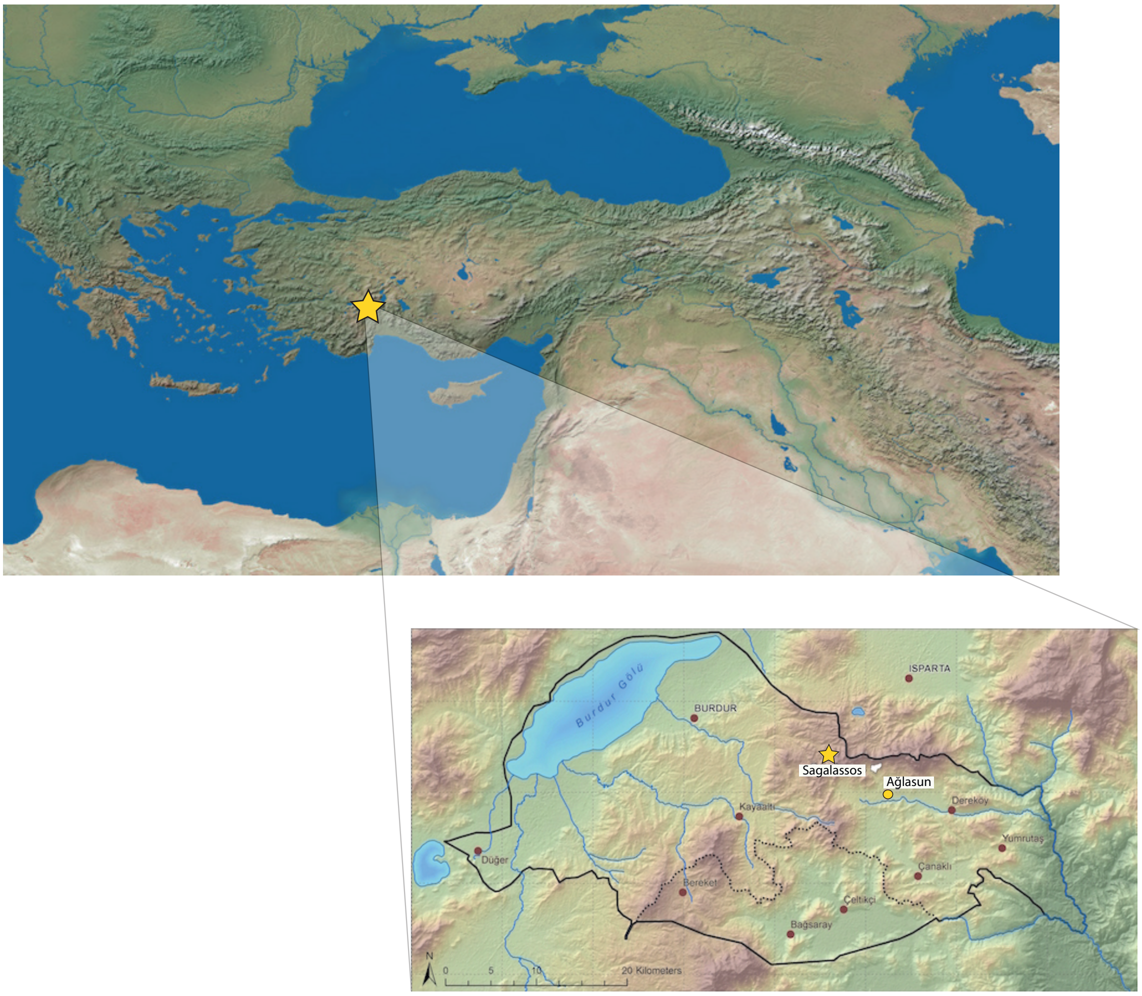

Figure S1. Location of Sagalassos and detailed map of the study region.

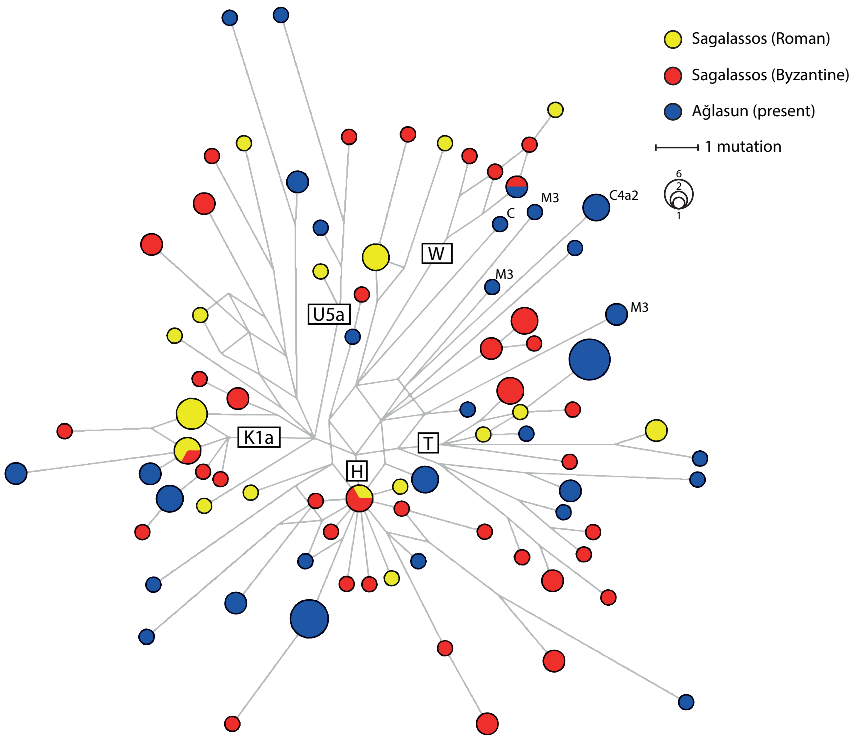

Figure S2. Median-Joining network of concatenated HVS1-HVS2 haplotypes of Sagalassos (Roman and Byzantine) and Ağlasun. Node size is proportional to the number of haplotypes as reported in the legend.

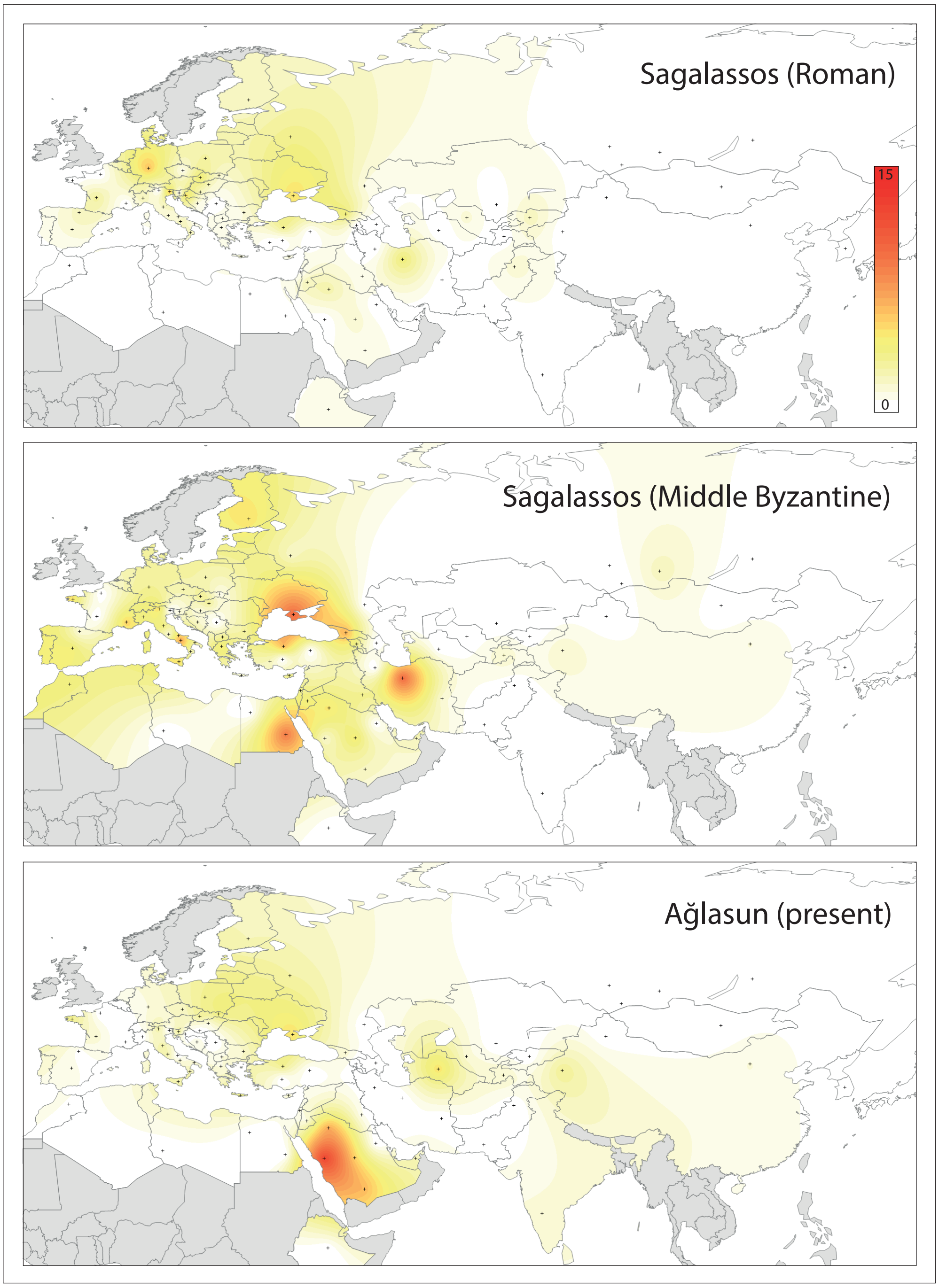

Figure S3. Contour maps of relative frequencies of concatenated HVS-1 and HVS-2 haplotypes from Sagalassos and Ağlasun in 77 modern populations of the database, after excluding the CRS haplotype that is widespread in west Eurasia. Haplotype frequencies are represented by a gradient from red (higher frequencies) to white (lower frequencies).

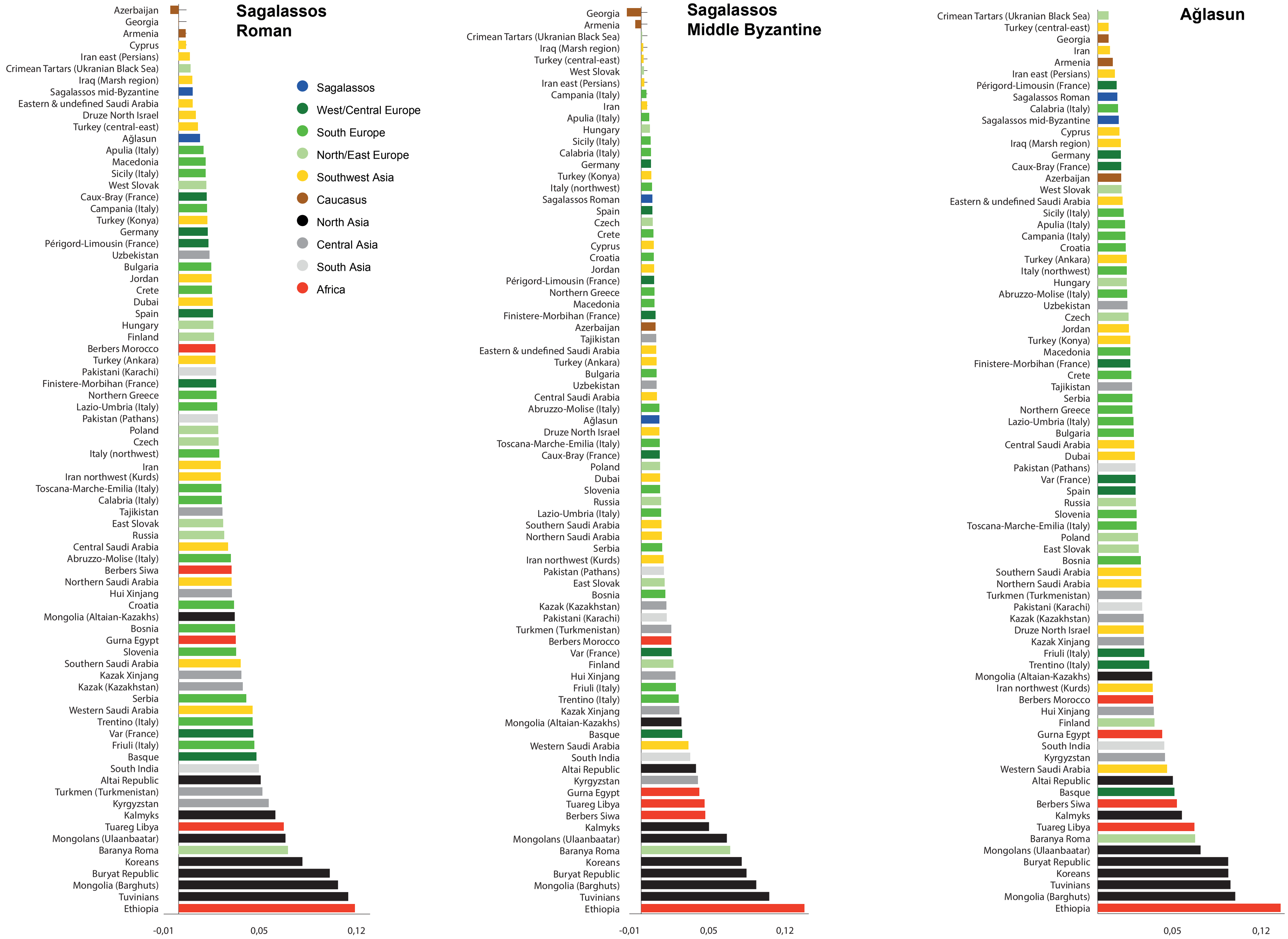

Figure S4a. Histogram of F_{ST} values of Sagalassos (Roman and Middle Byzantine) against the populations in the database of HVS1-HVS2 sequences (Table S4). The outlier Chukchi sample was removed from the analysis.

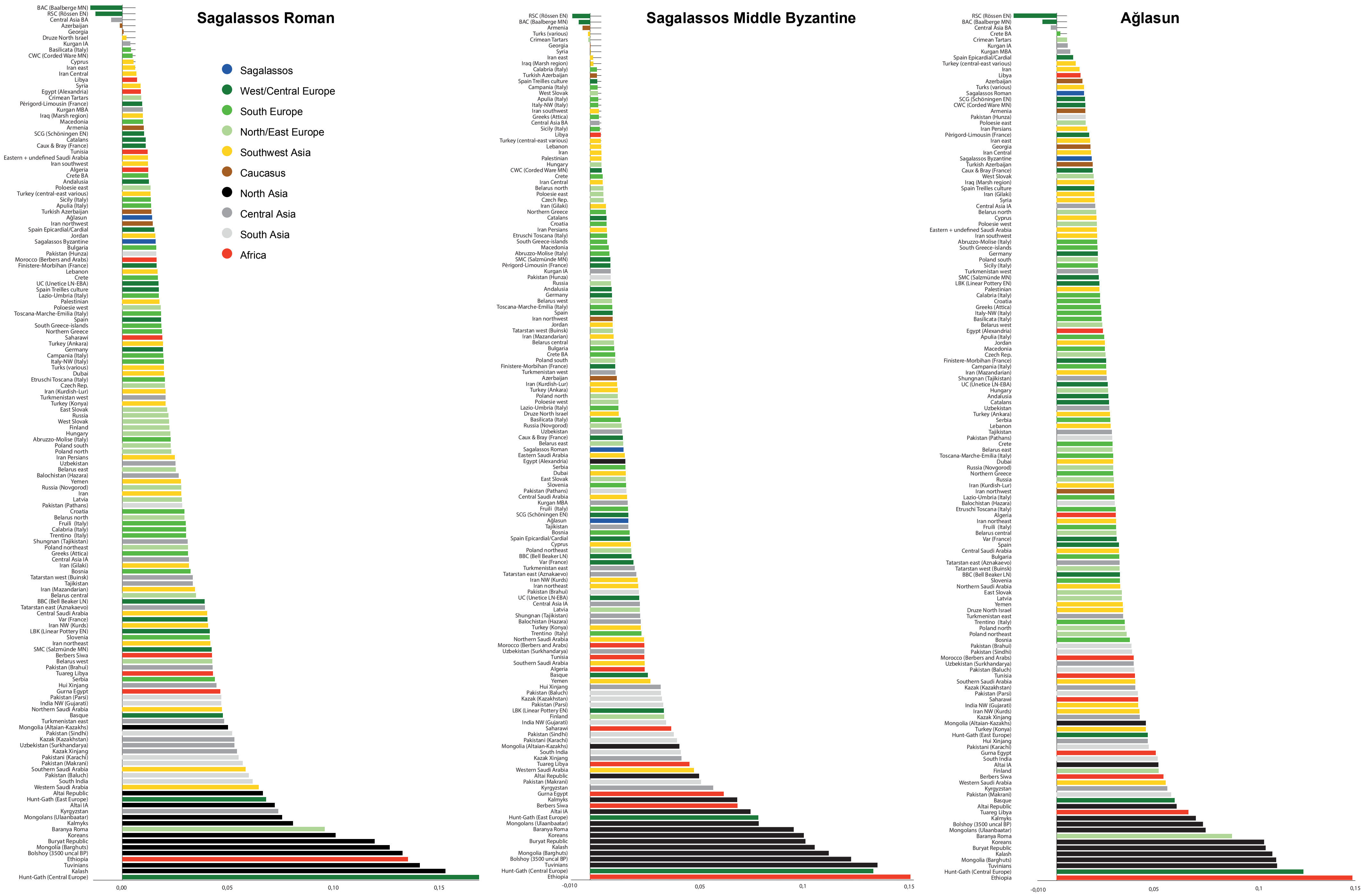

Figure S4b. Histogram of F_{ST} values of Sagalassos (Roman and Middle Byzantine) against the populations in the database of HVS1 sequences (Table S4). The outlier Chukchi sample was removed from the analysis.

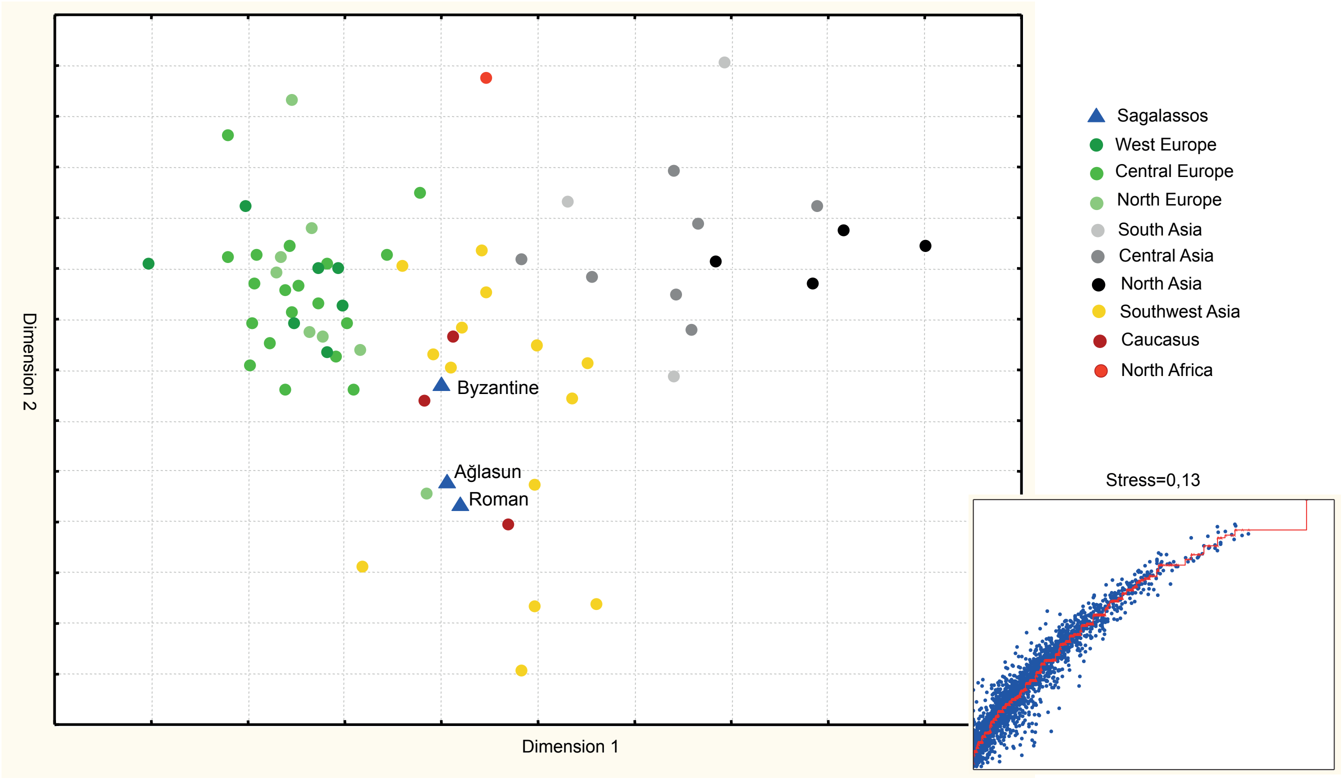

Figure S5. Two-dimensional MDS plot of Fst genetic distances estimated from the database of concatenated HVS-1 and HVS-2 sequences (see table S3).

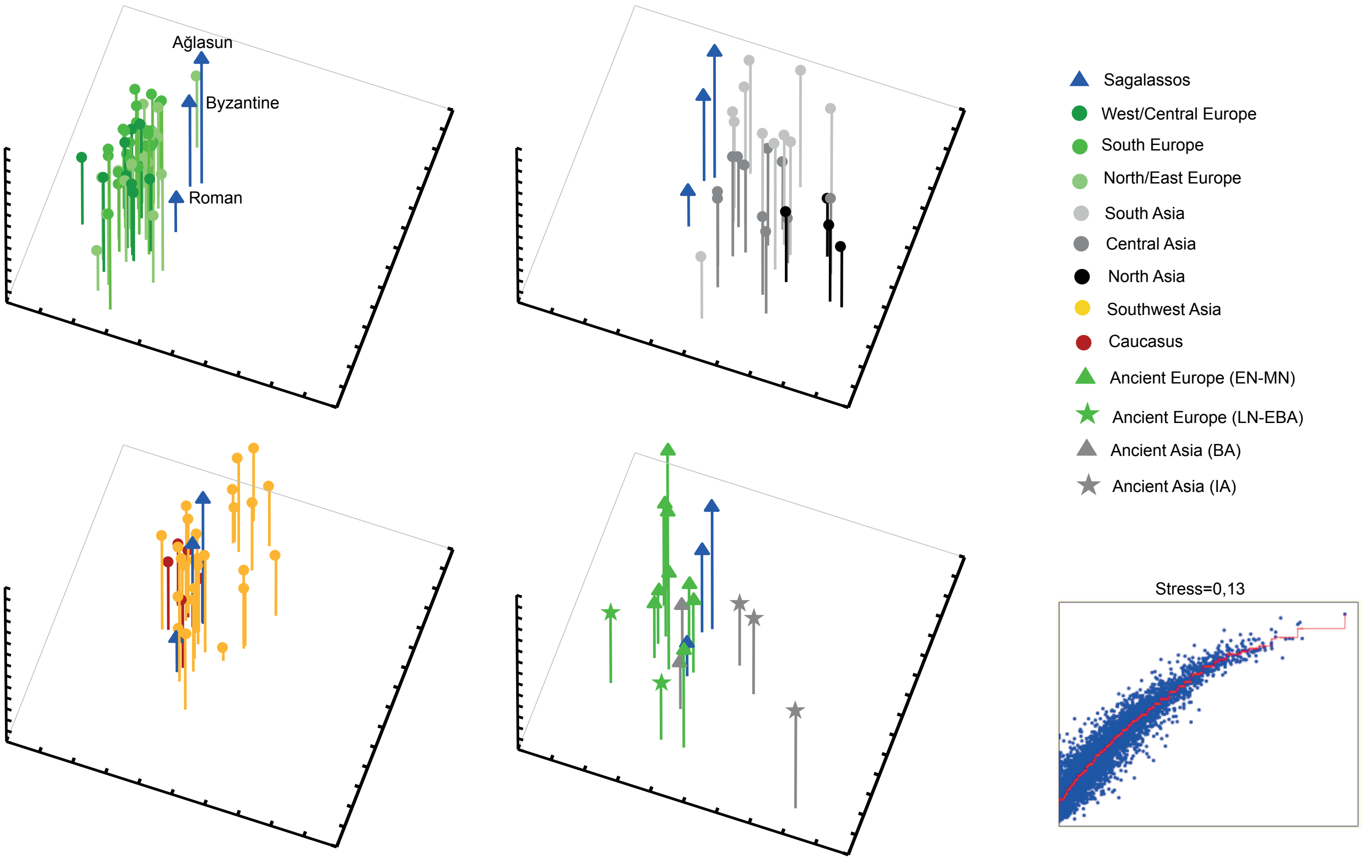

Figure S6. Three-dimensional MDS plot of Fst genetic distances estimated from the database of HVS-1 sequences (see table S4).

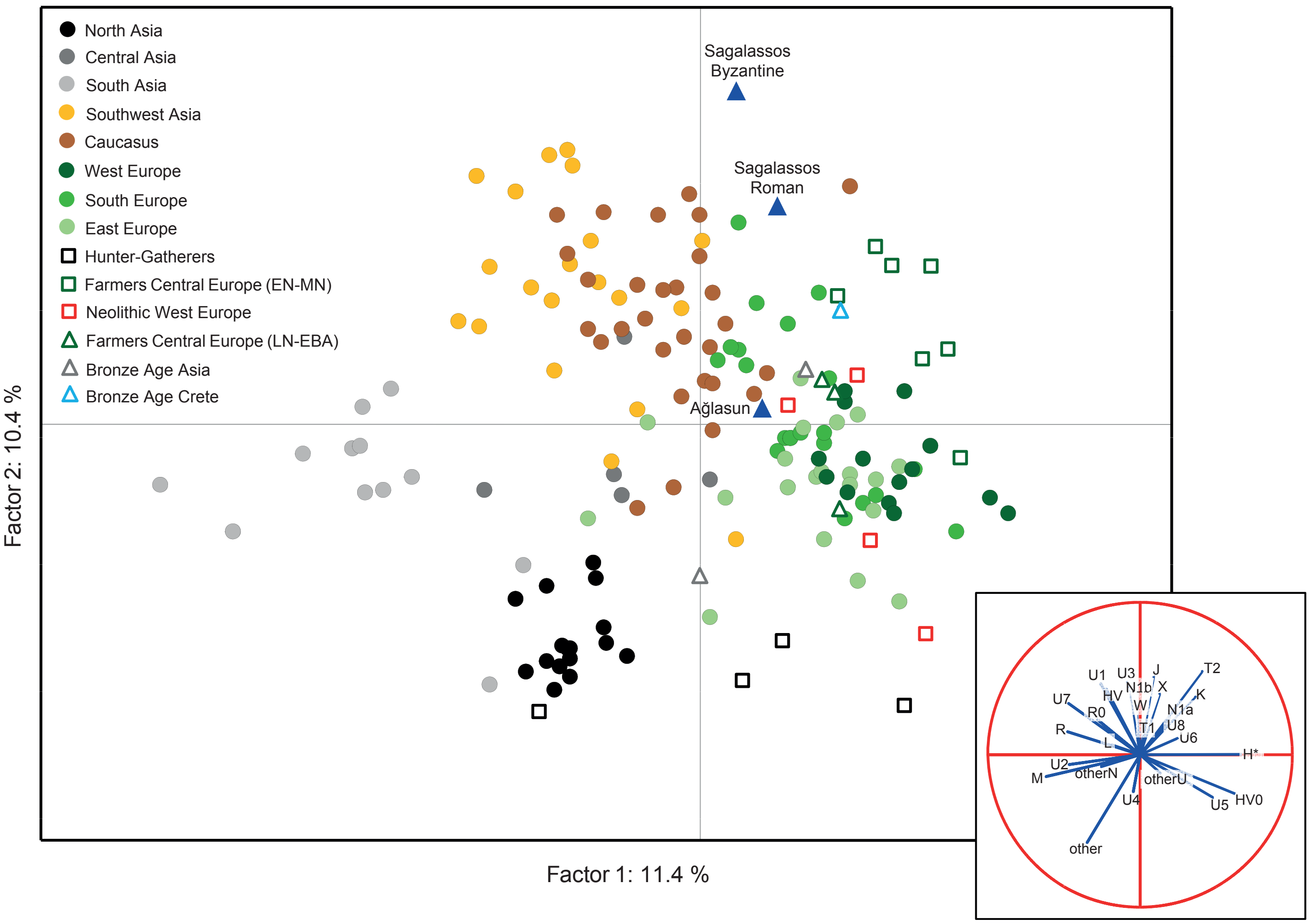

Figure S7. Two-dimensional plot of the PCA of haplogroup frequencies of modern and ancient populations of the database (see table S9).

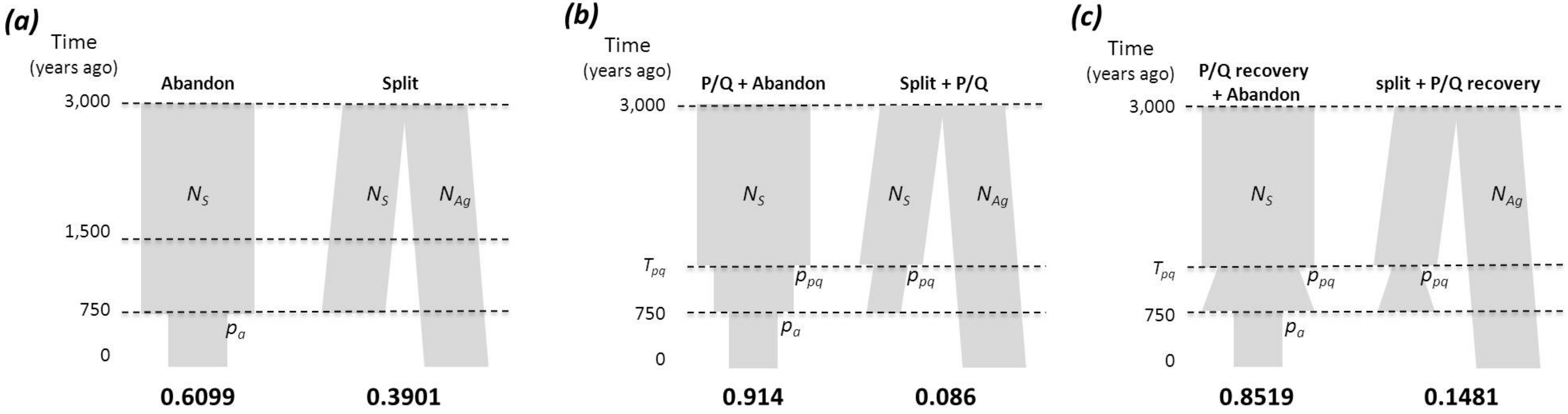

Figure S8. Continuity *vs* discontinuity (split) ABC model selection. We applied ABC model selection to three sets of continuity *vs* discontinuity scenarios. For each of the three continuity scenarios described in Figure 1B, we built the correspondent discontinuity one assuming isolation between the populations of the city of Sagalassos and Ağlasun, since their split 3,000 years ago. In these new scenarios, we assume different effective population sizes for Sagalassos and Ağlasun (N_S and N_{Ag}, respectively; see supplementary text table 1) and a complete disappearance of the Sagalassos population 750 years ago, corresponding to the abandon of the city. In all the three sets analysed, whether we model the abandon of the city like in (a) or we add complexity and assume a bottleneck in Sagalassos corresponding to the plague/quake, with (c) or without (b) subsequent recovery in population size, the values of the posterior probabilities (below each scenario) are always higher for the continuity scenarios. For further details on the parameters used, see section 2.1 and Figure 1.
