# Supplementary material for: Comparing maternal genetic variation across two millennia reveals the demographic history of an ancient human population in southwest Turkey
Source: R Soc Open Sci. 2016 Feb 17;3(2):150250. doi: 10.1098/rsos.150250 (PMC4785964; doi:10.1098/rsos.150250)
Supplement: 1 supplementary text file with a detailed description of the methods used in the present study. [file rsos150250supp3.docx]

**Supplementary text**

**Comparing maternal genetic variation across two millennia reveals the demographic history of an ancient human population in Southwest Turkey.**

Claudio Ottoni^1,2,3^, Rita Rasteiro^4^, Rinse Willet^5^, Johan Claeys^6^, Peter Talloen^6^, Katrien Van de Vijver^1^, Lounès Chikhi^7,8,9^, Jeroen Poblome^6^, Ronny Decorte^2,3^.

^1^ Center for Archaeological Sciences, University of Leuven, Leuven, Belgium.

^2^ Laboratory of Forensic Genetics and Molecular Archaeology, UZ Leuven, Belgium.

^3^ Dept. of Imaging & Pathology, University of Leuven, Leuven, Belgium

^4^ Dept. of Genetics, School of History, University of Leicester, Leicester, UK

^5^ Dept. of Humanities, Institute of History, Leiden University, The Netherlands

^6^ Sagalassos Archaeological Research project, University of Leuven, Belgium

^7^ CNRS, Université Paul Sabatier, ENFA; UMR5174 EDB (Laboratoire Évolution & Diversité Biologique), Toulouse, France

^8^ Université Toulouse 3 Paul Sabatier, CNRS ; UMR5174 EDB, Toulouse, France

^9^ Instituto Gulbenkian de Ciência, Oeiras, Portugal

**1. Laboratory methods**

**1.1. Precautions taken to minimise contamination**

The genetic analyses were performed in the Laboratory of Forensic Genetics and Molecular Archaeology in Leuven (Department of Imaging and Pathology, University of Leuven, Belgium), which is equipped with dedicated pre-PCR ancient DNA (aDNA) facilities physically separated from post-PCR facilities. The aDNA facilities were routinely cleaned with bleach and RNAse Away (Molecular BioProducts, San Diego, CA, USA) and every item entering the room was extensively washed with bleach or RNAse Away and when possible UV-irradiated. Buffer, MgCl_2_ and BSA used for preparation of amplification reactions were UV-irradiated to minimize the risk of contamination from reagents [1, 2] and autoclaved nuclease-free water (Promega) was used. In addition, carry-over prevention strategies by means of uracil-N-glycosylase (UNG) were adopted [3].

Access to the pre-PCR laboratory was restricted to a limited number of people and only after wearing clean overalls, gloves, over-shoes, surgical facemasks, plastic spectacles, and following an irreversible sequence of work steps to avoid contamination. Access to the pre-PCR lab was not permitted if PCR products had been handled the same day.

**1.2. Preparation of samples, DNA extraction and amplification**

To extract DNA from teeth and bones, one sample was prepared at a time. The outer surface of bone and teeth samples was removed through sterile blades or with a Dremel drill (Dremel, Racine, WI, USA). The surface of the teeth were gently wiped with a dilute solution of bleach (5-10%, wt/vol) and rinsed with bidistilled water. Bone and teeth samples were subsequently ground into a fine powder in a 6750 Freezer Mill (SPEX CertiPrep, Metuchen, NJ, USA) and stored at 4°C.

Hydroxylapatite powder was processed in parallel with the bone and teeth samples as a blank control to check for contamination during the grinding step in the Freezer Mill vials.

Aliquots of 0.2-0.4 g bone and tooth powder were incubated for 24-48h at 37°C on a rotating wheel in 1.8 mL digestion solution of 0.5 M EDTA pH 8 (Invitrogen, Carlsbad, CA, USA) and 0.25 mg/mL proteinase K (Roche, Penzberg, Germany). DNA was purified following a protocol based on the QIAquick Gel Extraction kit (Qiagen, Hilden, Germany) and with a vacuum manifold (Qiagen) using 15 mL tube extenders (Qiagen). Final DNA elution was done in two steps, each using 30 μL EB elution buffer (Qiagen) heated to 65°C. Each independent extraction batch contained one blank control every five archaeological samples.

Analysis of the first and the second hypervariable segments (HVS-I and HVS-II) of the mtDNA control region was performed by amplification in singleplex and direct sequencing, respectively, of five and two overlapping fragments (ranging from 109–166 bp in size), in order to read 357 bp in the HVS-I, from nucleotide position (np) 16009–16365, and 217 bp in the HVS-II, from np 49–265, as described in Ottoni *et al.* [4]. Amplification of the nine fragments in the mtDNA were performed in a final volume of 50 μL containing 1x PCR Gold Buffer (Applied Biosystems), 2.5 mM MgCl2 (Applied Biosystems), 0.2 mM each of dGTP, dATP, dCTP, 0.4 mM of dUTP (Bioline), 0.05-0.1 µM each primer (Eurogentec, Seraing, Belgium – IDT, Leuven, Belgium), 0.05 % BSA (Sigma Aldrich, St. Louis, MO, USA), 0.5 units of Uracil-DNA glycosylase (UNG, ArcticZymes) and 2.5 U AmpliTaq Gold® DNA polymerase (Applied Biosystems), 5-8 µL of aDNA extract. The following cycle conditions were used: 37°C for 15 min, 94 °C for 10 min, 45 cycles of 94 °C for 45 sec, 56 °C for 1 min, 72 °C for 1 min, and a final step of 72 °C for 5 min.

Analysis of the eight SNPs in the mtDNA coding region was performed by amplifying in multiplex eight fragments ranging in size 102-134 bp, followed by minisequencing reaction. All primers and PCR conditions used are as in Ottoni *et al* [4].

**1.3. Preservation of aDNA in Sagalassos and authentication of data.**

Preservation of aDNA in the archaeological site of Sagalassos has already been assessed in previous works in archaeological samples of humans [4], pigs [5], fish [6] and cats (study in preparation) as old as the human samples analysed in this study (Classical/Roman time).

Archaeological samples (teeth and bones) to be submitted to aDNA analyses were taken from the skeletons *in-situ* by an anthropologist (KV) and secured from further human handling and analysis until they were sent to the laboratory of Molecular Archaeology of Leuven.

Ancient DNA sequences of the archaeological specimens were considered authentic only when reproduced across multiple independent extractions (minimum two and up to three) and amplifications (minimum two and up to four in each extract). The sequences observed did not match any of those of technicians and researchers involved in the excavations and in the genetic analyses. In seven individuals no DNA amplification out two independent extractions was obtained. Samples with low amplification yields which provided ambiguous haplotypes, or which matched sequences observed in the blank controls, were discarded (13 out of 44 specimens).

A large number of blank controls were analysed in parallel with the archaeological samples at every analytical step, from grinding up to amplification reaction set-up. A total of 855 bank controls were tested, of which 145 (17%) were positively amplified and sequenced. In no instance sequences from blank controls matched those of the authenticated archaeological samples, or were reproduced over multiple extraction and amplification batches across different time points. Despite some reagents being decontaminated by UV-irradiation (PCR-buffers, BSA) we believe that such background contamination was present in commercial reagents [2] (e.g. primers, extraction buffers) and plastic-ware, and only in few instances (<1%) was introduced by the operator involved in the genetic analyses (CO).

All sequences from the archaeological samples were validated and considered authentic after a Fisher’s exact test with a >95% confidence level [2] used to determine whether the PCR success rate of the archaeological samples is significantly different from the amplification rate due to potential contaminants in the reagents [1].

Polymorphic sites observed in the authenticated individuals were always 100% consistent across multiple independent amplifications. No singleton mutations were observed, most likely due to the fact that the use of UNG destroyed damaged DNA fragments with cytosines deaminated *post-mortem*.

Authenticity of our aDNA data is further attested by the fact that the archaeological site of Sagalassos is located at altitudes between 1,450 and 1,600 m in the Taurus mountain range. The climate diagram of the nearest city (Isparta, at an altitude of about 1,000 m) indicates a mean annual temperature of 12 °C [7]. Using the best available approximation for the temperature-dependency of mtDNA decay in bone based on depurination kinetics [8], we estimated the rate of amplifiable DNA loss as a function of time and temperature. The fraction of remaining 166 bp-long amplifiable DNA molecules (the longest fragment that we amplified) in 1,800 years old samples was estimated as 27 % (with a molecular half-life of 957 years for the targeted 166 bp fragment). This suggests that temperatures in Sagalassos are likely to be compatible with the survival of amplifiable DNA in the 2^nd^-4^th^ century CE Roman samples analysed.

The mtDNA sequences reported in this study reflected polymorphisms overall congruent with the geographic location under study. No sequences showed obvious conflict with haplogroup-defining segregating sites. The phylogenetic consistency between sequences from different chronological periods – Roman Sagalassos, Middle Byzantine Sagalassos and Ağlasun – produced independently at different time points strongly indicate that the observed data are authentic.

Overall, the strict laboratory procedures followed to analyse the archaeological samples together with the authentication criteria adopted make it highly unlikely that the haplotypes observed in our samples arose from contamination or *post-mortem* damage.

**1.4. Data Analyses**

The haplotype of each individual was assigned to a haplogroup according to the latest mtDNA tree [9] (<http://www.phylotree.org/>). When possible, haplogroup assignations were also done for 53 mtDNA control region sequences previously reported from the town of Ağlasun [10].

To unravel the genetic affinities of two ancient samples from Sagalassos (Roman and Middle Byzantine) and the modern sample from Ağlasun to present-day populations, we generated an in-house database with more than 11,000 concatenated HVS-1 and HVS-2 mtDNA sequences of contemporary populations from the literature. Pairwise F_ST_ genetic distances were computed with Arlequin 3.5 [11] and significant variations in their values were tested by means of 10,000 permutations. P-values were adjusted to correct for multiple comparisons with the Benjamini-Hochberg method [12] using the function *p.adjust* in R [13].

In order to visualise the pattern of genetic differentiation of the two ancient samples from Sagalassos and the modern one from Ağlasun with present-day populations, Slatkin’s linearized F_ST_ values were plotted on a geographic map with Surfer 6 (Golden Software) using the Kriging method. To further explore the spatial distribution of genetic differentiation the Slatkin’s F_ST_ values were represented in a two-dimensional Multidimensional Scaling (MDS) plot with Statistica ver 8 (Statsoft).

Haplotypes from Sagalassos and Ağlasun shared with the samples of the database were found with Arlequin 3.5. The relative frequencies of shared haplotypes in the modern populations were displayed on a geographic map with Surfer, after excluding the CRS haplotype that is widespread in west Eurasia. A median-joining network [14] of concatenated HVS1-HVS2 haplotypes was created with Network v4.6 (http://www.fluxus-engineering.com) to visualize the relationship between the ancient samples of Sagalassos (Roman and Middle Byzantine) and the modern village of Ağlasun.

To include a larger number of populations as well as ancient samples from the literature, a second database of more than 17,000 HVS-1 sequences was used to compute F_ST_ genetic distances, which were graphically plotted in a three-dimensional MDS as described above.

A Principal Component Analysis (PCA) was carried out with Statistica on the basis of haplogroup frequencies from more than 180 ancient and modern populations and represented in a two-dimensional plot. Detailed information about polymorphic sites in Sagalassos and Ağlasun, genetic distances, shared haplotypes, haplogroup frequencies, and the list of populations used for comparative analyses are in the electronic supplementary tables.

In order to assess whether the different sample size of the three samples analysed might have affected the pattern of F_ST_ genetic distances observed, we down-sampled the Byzantine sample and the Ağlasun sample to the size of the Roman sample (n=24) and repeated the calculation of F_ST_ genetic distances 10 times. The results were plot in a map with Surfer (Figure S9) and showed in all instances a higher degree of affinity (lower genetic distances) of the Byzantine sample with the modern populations, and a pattern of genetic affinity with the modern populations similar to that observed in the original non-downsampled data (except for one instance in the Ağlasun sample, which showed higher affinity to Asian populations).

**2. Approximate Bayesian Computation (ABC) analyses**

**2.1. Demographic scenarios**

Two sets of different demographic scenarios were tested using ancient and modern mtDNA data of Sagalassos and the nearby town of Ağlasun.

In the first set, we studied the impact of a single past population size contraction following either the Plague of Justinian (PM) or the 7^th^ century earthquake (QM) or the abandonment (AM) of the city of Sagalassos (see figure 1a and supplementary text table 1). These scenarios assume a single constant female effective population size *N_S_*, prior to the contraction, sampled from an ancestral female population of constant size *N_anc_*, corresponding to the initial settlers of the region. A scenario of no size change since the BO-phase in the region until the present in Ağlasun was also tested as a null hypothesis.

In the second set (see figure 1b), a second population size past contraction was added to the AM scenario. This population size contraction corresponds to a size reduction during the plague and/or quake at time *T_pq_* and was either followed by a period of recovery (PQR+AM) or constant population size (PQ+AM) until the abandonment of the city of Sagalassos.

**Supplementary text table 1.** Broad uniform prior distributions were used for the parameters shown in figure 1 and figure S8. The parameter *p_i_* (*p_p_*, *p_q_*, *p_a_* and *p_pq_* for plague, quake, abandonment of the city and *T_pq_*, respectively) represents the proportion used to calculate the effective population size after the contraction (*N_C_*) at time *i*, such that *N_C_ = p_i_×N_S_* for scenarios AM, PM and QM. In the more complex scenarios, *N_C_* after the abandonment of the city is *p_pq_×p_a_×N_S_* and *p_a_×N_S_* for PQ+AM and PQR+AM, respectively. The parameter *p_pq_* is informed by historical evidence that estimates that the Plague of Justinian have erased up to 30% of the Anatolian population [15]. The parameter *T_pq_* corresponds to a period of time that englobes both the Plague of Justinian and the 7^th^ century earthquake. *N_Ag_* parameter is described in section 2.3.

| **Parameter** | **Prior** |
| --- | --- |
| *T_pq_* (in generations) | U:50-65 |
| *p_pq_* | U:0.2-1 |
| *p_a_* | U:0.01-1 |
| *N_S_* | U:1,000-40,000 |
| *N_Ag_* | U:1,000-40,000 |
| *N_anc_* | U:1,000-5,000 |

**2.2. Approximate Bayesian Computations (ABC) for model selection and parameter estimation**

We used Bayesian Serial SimCoal [16, 17] to simulate aDNA and modern mtDNA data, by tracing the female ancestry of the Ağlasun (N=53) modern samples and incorporating ancient DNA samples of both Middle Byzantine (N=51, ranging temporally the 11^th^-13^th^ century CE) and Roman (N=22, ranging the 2^nd^-4^th^ century CE) periods. Chronologically, the two Early Byzantine samples (7^th^ century CE) are closer to the Roman sample, for this reason they were pooled to it in the comparative analyses with other populations of the database (F_ST_ genetic distances, haplotypes shared and PCA). However, in the Bayesian coalescent analysis, to keep consistency in the periodization of the samples used and a sharp temporal separation between two chronological groups before and after the bottleneck tested (plague and earthquake in the 6^th^-7^th^ century CE) we excluded the two Early Byzantine individuals.

The three sets of sequences were sampled from the coalescent simulations according to the sample sizes of the observed sequence data and their corresponding ages (Table S2). We generated a sequence of 574 bp, using the following parameters of sequence evolution for all simulations: a fixed mutation rate of 7.5x10^-6^ substitutions per site per generation [18], a transition bias of 0.9841 and a continuous gamma distribution of mutation rates among sites with shape parameter 0.205 [19] and a generation time of 25 years [20].

To determine which of the demographic models (described in the main text and above) explained better the data and to estimate the demographic parameters of interest we used an ABC approach [21, 22], based on the observed and simulated genetic summary statistics. We performed 1,000,000 simulations for each model and selected the 0.1% simulations that best explained the observed data.

The ABC inference procedure was performed using the *abc* R package [23]. The *postpr* function was used to select the best model. This was done using the Beaumont *et al.* [21] multinomial logistic regression (MLR) model and with a correction for heteroscedasticity. Parameters were estimated using the 0.1% simulations associated with the shortest Euclidian distances from the observed data. The ridge method that performs a local-linear regression and deals with collinearity issues, jointly with a logit transformation and also with a correction for heteroscedasticity, was used to estimate the parameters based on the observed and simulated summary statistics [22, 23].

The model selection approach was validated by calculating the power to recover the true model (Supplementary text tables 2 and 3). To do that, we took randomly 1,000 datasets from the original BayeSSC runs for ABC analysis, for each of the demographic models. We thus assigned each of these datasets to a demographic model, by using function *cvpostpr* also from the *abc* R package also retaining 0.1% simulation with the shortest Euclidian distances. However, this time we used the summary statistics values of the simulated datasets as pseudo-observed summary statistics. Finally, we counted the number of times that the true model was correctly identified.

**Supplementary text table 2. Validation of the ABC model selection procedure represented in figure 1a.** Each row corresponds to the percentage of times that a model (AM, CM or PM/QM) was assigned to each of the models, by a higher posterior probability. Both models PM and QM were pooled for the validation, as they are very similar and just differ by five generations for the time of contraction either referring to the plague or quake (see figure 1a).

|  | AM | CM | PM/QM |
| --- | --- | --- | --- |
| AM | 41.7 | 39.3 | 19 |
| CM | 19.7 | 57.1 | 23.2 |
| PM/QM | 19.7 | 38.1 | 42.2 |

**Supplementary text table 3. Validation of the ABC model selection procedure represented in figure 1b.** Each row corresponds to the percentage of times that a model (AM, PQ+AM or PQR+AM) was assigned to each of the models, by a higher posterior probability. Our simulations suggest that there is a bias favouring the AM model, and much less the PR+AM and PQR+AM models. One reason for this is that when simulating the plague/quake population contraction, the simulations retained by the ABC procedure have a very small contraction proportion that makes the three models be very similar between them.

|  | AM | PQ+AM | PQR+AM |
| --- | --- | --- | --- |
| AM | 47 | 25 | 28 |
| PQ+AM | 31 | 45.8 | 23.2 |
| PQR+AM | 38.6 | 28 | 33.4 |

**2.3. Testing for continuity *vs* discontinuity hypotheses**

Since our modern data could not be traced to the ancient samples, we applied two different methods to test for continuity versus discontinuity between the two ancient samples of Sagalassos (Roman and Middle Byzantine) and the modern sample of Ağlasun.

**2.3.1 Continuity model: rejection/acceptance test**

First, we applied a methodology similar to one described elsewhere [24]. We performed coalescent simulations under a continuity model that assumes that the modern samples of Ağlasun were descendants of the individuals that inhabited the region when Sagalassos was inhabited. This model assumes a single past population contraction following the abandonment of the city of Sagalassos and a single constant female effective population size *N_S_*, prior to the contraction, sampled from an ancestral female population of fixed constant size *N_anc_* (3000), corresponding to the initial settlers of the region. We explored 2,500 parameter combinations using fifty equally spaced values, sampled from the priors for both combination of *N_S_* and *p_a_* values (ranging from 1,000 to 40,000) and *p_a_* (ranging from 0.01 to 1), being *p_a_* the proportion used to calculate the effective population size after the contraction (between 10 and 40,000). For each pairwise combination we performed 500 independent coalescent simulations, hence corresponding to a total of 1,250,000 simulations. We used Bayesian Serial SimCoal software (BayeSSC) [16, 17] to simulate aDNA and modern DNA data and used the same parameter values for sequence sizes, mutations rates, transition bias, distribution of mutations rate among sites and periods of time as described in section 2.2. Three sets of sequences were sampled from the coalescent simulations according to the sizes of the observed sequence data (Roman, Byzantine and Ağlasun) and their corresponding ages. We then computed the pairwise F_ST_ values in the simulated data and compared them to the values observed in the real data. The proportion of times where the simulated F_ST_ was greater than the observed F_ST_ was recorded, for each combination of *N_S_* and *p_a_* values. We also computed whether the observed F_ST_ values were within the 95% confidence interval for each parameter combination. Scripts were written in the R language [10] to create the infiles read by BayeSSC and to analyse the results. The 2,500 points forming the grid and for which the probabilities were estimated, were used to produce interpolated plots with the filled.contour R function (data not shown).

For each of the 2,500 parameter combination, all the observed F_ST_ values were within the 95% confidence interval. For all the combination of parameters and for all the populations compared, the estimated probability of obtaining *F_ST_* values that are equal or higher than those observed in the real data (P_S>O_) was always higher than a p-value of 0.05. In fact, P_S>O_ can reach values of 0.9 for the Roman vs Byzantine and Roman vs Ağlasun comparisons. Our results show that the parameter space appears to explain the observed data, i.e. the continuity hypothesis is not rejected.

**2.3.2 Continuity *vs* discontinuity models: ABC test**

In the second method, we built new scenarios based on the ones described in figure 1b, but assuming that the modern population of Ağlasun did not share any demographic past with the city of Sagalassos, since their settlement in the Ağlasun valley 3,000 years ago. We called these new scenarios discontinuity or split models (see figure S8). These models assume different effective population sizes for Sagalassos and Ağlasun (N_S_ and N_Ag_, respectively; see supplementary text table 1) and a complete disappearance of the Sagalassos population 750 years ago, corresponding to the abandonment of the city.

We then applied the same ABC procedure described in section 2.2 to each of the models described in figure 1b, but this time we made pairwise comparisons to their correspondent discontinuity model. Our results show that models assuming continuity between modern and ancient samples are the ones with a higher posterior probability of being selected (see figure S8 and validation in Supplementary text table 4) and that is why we decided to use continuity models in our analyses (see main text).

**Supplementary text table 4. Validation of the ABC model selection procedure for the three sets of continuity *vs* discontinuity scenarios represented in figure S8.** In each panel, each row corresponds to the percentage of times that a model was assigned to each of the two models, by a higher posterior probability. For details of validation procedure see section 2.2

|  | Abandon | Split |
| --- | --- | --- |
| Abandon | 46.9 | 53.1 |
| Split | 26.5 | 73.5 |
|  | P/Q + Abandon | Split + P/Q |
| P/Q + Abandon | 78.1 | 21.9 |
| Split + P/Q | 38.8 | 61.2 |
|  | P/Q recovery + Abandon | Split + P/Q recovery |
| P/Q recovery+Abandon | 72.7 | 27.3 |
| Split + P/Q recovery | 50.1 | 49.9 |

**2.4. Summary Statistics**

In our ABC approach, we calculated several measures of genetic diversity to summarize our observed data (see supplementary text tables 5, 6 and 7), using Arlequin ver. 3.5.1.2 [11]. The same statistics were also calculated for our simulated data by BayeSSC program.

The within population summary statistics calculated for each population are the following: number of haplotypes (K), number of segregating sites (S), gene diversity (H_e_), nucleotide diversity and Tajima’s D. Between populations’ summary statistics included Hudson’s F_ST_ [25] and number of haplotypes private to each population (as defined in supplementary text table 7).

**Supplementary text table 5.** Within-population genetic diversity summary statistics for each of the samples studied (N, K and H_e_ correspond to sample size, number of segregating sites and gene diversity, respectively).

| **Summary Statistics** | **Roman (N=22)** | **Byzantine (N=51)** | **Ağlasun (N=53)** |
| --- | --- | --- | --- |
| **K** | 15 | 39 | 30 |
| **S** | 42 | 71 | 81 |
| **Nucleotide diversity** | 0.013 | 0.0154 | 0.017 |
| **H_e_** | 0.909 | 0.970 | 0.945 |
| **Tajima’s D** | -1.372 | -1.547 | -1.520 |

**Supplementary text table 6.** Population genetic differentiation measured by pairwise F_ST_ values.

| **F_ST_** | **Roman** | **Byzantine** |
| --- | --- | --- |
| **Roman** | - | - |
| **Byzantine** | 0.015 | - |
| **Ağlasun** | 0.025 | 0.016 |

**Supplementary text table 7.** Number of haplotypes private to each population (*P_ij_*) in pairwise population comparisons. The table should be read as following: number of haplotypes private to population *i* (in the first column) when compared with population *j* (first row).

| **P_ij_** | | **Roman** | **Byzantine** | **Ağlasun** |
| --- | --- | --- | --- | --- |
|  |  | ***j*** | | |
| **Roman** | ***i*** | - | 13 | 15 |
| **Byzantine** |  | 37 | - | 38 |
| **Ağlasun** |  | 30 | 23 | - |

**2.5 Replication of simulations assuming potential maternal relationships.**

Whereas potential maternal relationships could be addressed and were accounted for in the Byzantine sample [4], in the Roman samples the only factor suggesting family relationships was the spatial proximity of the burials within the PQ4 compound. In the absence of clear evidence of direct maternal relationship (as also corroborated by the anthropological survey, see section 3.1) we considered all haplotypes in the Roman sample as unrelated.

However, in order to test whether maternal relationship could affect our results, we repeated the coalescent simulations, described in figure 1a, after collapsing all potentially related individuals based on the spatial proximity of the burials. Nine individuals were reduced to three, namely KV03-84-35-11 assigned to haplogroup K1a, KV17-74 assigned to T1a1’3, KV21-24-28 assigned to X2b. We recalculated the observed summary statistics and reapplied the ABC framework described in section 2.2. The posterior probabilities found were very similar to the ones presented in figure 1a (e.g. the AM city-abandonment model had a posterior probability value of 0.9341), thus making us confident that our results were not affected by a possible kinship.

Further analysis of high-resolution nuclear markers may help address potential relationships among the individuals analysed here.

**3. Archaeological and anthropological context**

**3.1. Anthropological survey**

The burials, from which the samples used in the present study were taken, were studied between 2012 and 2014 to analyse the biological features of the remains through morphological and metric studies. The remains derive from five sites, dated between the mid-4^th^ century BCE and the 7^th^ century CE.

Eleven burials were recorded in 2012 at Site F in Eastern Suburbia, eight inhumations and three cremations. The inhumation burials consisted of two built tombs, one double collective burial (tomb 1), besides single pit burials with indications for a container. The cremations consisted of a secondary deposition, an urn burial and a primary cremation. The inhumations showed extensive surface abrasion, with generally limited disturbance. The individuals were estimated as adult, both male and female. All inhumation burials and the primary cremation were sampled.

Potter’s Quarter 1, a Naiskos tomb in Eastern Suburbia, consisted of a central and three adjoining chambers. The western chamber was almost empty while the central chamber contained two poorly preserved coffin burials, collected as three concentrations. The eastern chamber was used over a period of time and contained the disturbed remains of minimum four individuals, with indications for primary and secondary deposition. Preservation and disturbance hindered analysis, but age at death was estimated as adult for all individuals. Sex estimation for the disturbed remains resulted in male individuals and individuals of indeterminate sex.

Another burial site from Eastern Suburbia was Potter’s Quarter 4, a walled enclosure. Twenty inhumations, two built graves and 18 coffin burials, were recorded. One coffin contained the remains of an adult female and the disturbed bones of a neonate. Preservation of the remains varied from relatively good preservation to severe surface abrasion and incompleteness. Age estimation ranged between neonatal and over 40 years old at death, both male and female individuals were recorded. All except two individuals, the neonate from the double burial and a disturbed adolescent, were analysed in the present study.

During excavations at an Early Byzantine church in the Southern Necropolis, Çatal Oluk 2, three *alla cappuccino* burials were recorded to the south of the building. The burials were incomplete with limited surface abrasion, and belonged to two adults and one child. Samples were taken for one adult, other samples belonged to disturbed remains.

The oldest burial was excavated at Tepe Düzen between two occupation layers in a workshop, dated in the mid-4^th^ century BCE. The grave was a plain earth burial and the poorly preserved remains belonged to an adult, estimated as male. Four other samples from Tepe Düzen were stray finds.

Study of the human remains shows that the use of the different burial sites varied. The different demographic composition may indicate the burial of different segments of society. Preservation varied with both relatively well preserved and poorly preserved, incomplete remains. In three contexts a relationship between individuals is indicated, in the collective burial in Site F, the burial of an adult female with a neonate in Potter’s Quarter 4, and in the Naiskos tomb. It was not possible to suggest family relationships based on the skeletal study. Further analyses in the future, including the analysis of nuclear markers, will help to assess potential kinship among the individuals, particularly in PQ4.

**3.2. Excavations at PQ4**

Twenty-five individuals unearthed from the Potters’ Quarter area were submitted to genetic analyses, of which 16 were successful, all from the site called PQ4.

A quadrangular structure of ca. 15 (N-S) by 21.5 m (E-W), built of polygonal blocks in dry masonry and situated to the east of the Potters’ Quarter, was selected for excavation in 2012 in order to determine its date and function. The discovery of twenty burials inside the eastern half of the walled enclosure identified it as a funerary compound of the Roman Imperial period.

Towards the end of the 1^st^ century CE, two cist-shaped inhumation tombs (n° 1 and 6) belonging to individuals estimated as female, probably members of the middle class judging by the associated grave goods, were built there. These tombs appear to have been at the origin of the compound. Not only were they the oldest burials, the north and east walls of the structure were also planned according to their position. Within the walled area, a floor level was subsequently laid out during the early 2^nd^ century CE using waste material from the potters’ workshops, and a partition wall was built to create different spaces within the complex.

After its completion, the burial compound had two main phases of usage, dating to the early 2^nd^ century CE (tomb n° 2) and the late 3^rd^ - 4^th^ century CE (tombs n° 3-5 and 7-20) respectively. All these burials were simple pit burials of individuals, mostly oriented to the east (though a western and northern orientation were also recorded) and using a wooden coffin for the entombment of the deceased. The funerary gifts, including coins, ceramic and glass unguentaria, and ornaments, were placed inside these coffins. The age composition of the cemetery was varied, including not only adult men and women, but also adolescents, children and even a neonate.

The absence of burials or material evidence dating to the 5^th^ century CE suggests a phase of abandonment, followed by a seismic event that caused wall blocks to fall into the complex. Afterwards, during the first half of the 6^th^ century, the compound was filled in with waste of nearby stone carving activity, and thus was sealed preventing from any further use.

**3.3. Excavations at Çatal Oluk 2**

The site is located on the more gentle slopes south of the city centre of Sagalassos. Field survey results originally identified the building as a suburban villa, but excavations in 2011 revealed the outlines of an Early Byzantine church measuring 25.9 m (west-east) by 15.8 m (north-south). The construction of the church could be dated to the late 5^th^ or early 6^th^ century CE, while the site was abandoned in the 7^th^ century CE. The southeastern quarter of the church was excavated, exposing the diakonikon (a small chamber lying in the axis of the southern nave) and parts of the three-naved naos and the bèma (containing the altar).

A small area outside the church (measuring 7 by 3 m) was excavated as well, in which the remains of three in situ tombs were encountered, dated to the 6^th^ or early 7^th^ century CE. All burials were cappuccina tombs, which meant that the inhumations were covered by large tiles in the shape of a thatched roof. Two of the burials only had their lower legs preserved in situ, as a deep pit was dug through them in order to accommodate two pythoi. The third burial, belonging to a child, was encountered unperturbed, but the fragile human remains were badly preserved. Throughout the surrounding and superimposing layers several loose skeletal fragments of human bodies were encountered as well, some of which were selected for DNA research.

**3.4. Excavations at Site F**

This site is situated on the northern slopes of the eastern artisanal-funerary proasteion of Sagalassos, generally referred to as the city’s Eastern Suburbium. It was the subject of excavations in 1990-1991 and has in more recent years (2011-2012) been reopened and extended as part of the wider research on this proasteion.

The steep slopes were subdivided in terraces from Classical times onwards, probably for agricultural reasons originally. Four excavation campaigns exposed a total area of 398 m², covering five subsequent terraces. These gradually became occupied with burials from Hellenistic times onwards till Late Roman times. A wide variety of burial types has been attested on the site: terracotta urns and a monumental aedicula tomb in Hellenistic times, a bustum burial dating back to Late Hellenistic times (reign of Amyntas), an Early Imperial vaulted family and, eventually, several individual pit burials and individual constructed tombs dating to Late Roman times. One wall perpendicular to the upper terrace wall furthermore contained an empty niche that could have been used as an ossuary. The archaeological data suggests that the terraces were subdivided into individual plots, but further research might shed more light on this aspect (a new excavation campaign is planned for the 2016 season).

The lower terraces were not uniquely in use as burial grounds, as we documented the erection of at least one Imperial pottery workshop, which was abandoned before the 3^rd^ century CE. Large parts of the terraces were subsequently used as dumping grounds for misfired pottery and the terraces would eventually be covered by erosional screes, providing good conservation conditions for the archaeological features underneath.

**3.5. Excavations at PQ1**

This site is located at the foot of the eastern slope of the Eastern Suburbium, the artisanal-funerary proasteion northeast of the city. The site displays both artisanal and funerary infrastructure: an Early Imperial pottery workshop, a 2^nd^ century CE temple-shaped naiskos tomb and a Late Roman pottery workshop. Each phase at least partially overlaps with one of the previous construction phases. The site has been excavated between 1998 and 2000 (with the focus on the workshops) and again in 2012-2013 (with the focus on the naiskos tomb).

The standing walls of the naiskos were only partially preserved, the two columns that stood between the antae were missing and there were no traces of the sarcophagi that once presumable stood on top of the benches inside the three niches along the cella walls. Nevertheless, the podium of the tomb, constructed of mortared limestone ashlars and rubble, was completely preserved. This podium still stood c. 2.10 m high and contained four hypogea: one larger central burial room and three individual rooms underneath each bench. The individual burial rooms could be entered from the cella; two of which were looted in ancient times.

In the eastern hypogeum the disturbed remains of three adult individuals could be recovered. The central and northern burials room were intact, and in the central one the scattered remains (scavenging animals) of two more individuals could be documented and recovered. The finds that could be recovered from the eastern and central hypogeum suggest that the naiskos was reused (usurped?) in the 5^th^ century CE. The northern hypogeum remains untouched to this day. No sample from PQ1 provided successful aDNA analyses.

**3.6. Demographic reconstructions**

Reconstructing ancient population levels is notoriously difficult, as contemporary statistics are only sporadically available and provide at best only insights for parts of the population. Therefore archaeologists and historians have relied upon proxy-data to estimate population sizes for antiquity, which in its most recent applications have resulted in population-level ranges rather than pinpointing the exact number of inhabitants of a city [26, 27]. Although ancient textual sources can be indicative for the (relative) size of an ancient city, the urban surface area and the number of houses are archaeological proxies which have been used extensively for population reconstruction [28].

For Sagalassos, the surface area of the Imperial city measured about 37.5 hectares (ha) (demarcated by the surrounding necropoleis), or 31 ha if the Eastern suburbium, which was not a residential area, is excluded. Using population densities derived from deductions in other pre-industrial cities, scholars often rely on the total city surface area (in this case 37.5 ha), of which only a part is considered used for residential zoning (50-56 %) [26, 29], which is then multiplied by a population density range of 125-250 people / hectare (for Sagalassos, this results in 2,343-5,250 inhabitants). However, a recent assessment of a multitude of archaeological projects by Simon Price [30], suggests a lower density of 40-60 people/ha for unplanned towns and for planned cities 110 people/ha. At Sagalassos an orthogonal street-grid, more typical for planned cities founded *ex novo*, is absent. Perhaps the topography of the city’s location on a mountainside prohibited the implementation of a regular grid, but the long history of the city is just as likely an explanation for this absence. The Augustan expansion of the city towards the east on the other hand seems more regular. If it is assumed that Sagalassos grew more organically (and a lower population density is assumed), then the population levels are much lower, between 1,500-2,250 inhabitants.

The archaeological evidence of the houses corroborates a lower figure: the Sagalassos Project recently inventoried the number of houses in a quarter of the city, which was explored using geophysical and archaeological prospection and partly by excavation. For an area of 8.7 ha, 92 houses were counted, which, extrapolated to the residential area of the entire city (some 31 ha) would equate to 328 houses. From tax records in Roman Egypt, it is known that the average size for a household of the lowest class in cities was between 4.04 and 5.31 persons [31]. At the 3^rd^ century BC city of Cosa (a planned Roman colony) in Italy, some 24 larger houses and 224 smaller houses were discovered, whereby the larger houses are assumed to have belonged to the local elite, which had a larger household of between 10-12 people [32, 33]. If this ratio is employed for Sagalassos, i.e. 30 larger houses for the elite (10-12 people/household) and 298 houses for the lower classes (4-5 people per household), the number of inhabitants ranges between 1,492-1,850. Although these two calculations match, it is necessary to remain cautious, as the excavations in the residential area of Sagalassos are still on-going. A slightly higher figure is possible, since other Roman cities, such as Pompeii, seem to have been dominated by elite housing [34]. Still, a figure below 5,000 inhabitants is highly probable as most of the cities of the Roman Empire seemed to have had similar population levels [27].

Another indicative factor for population of the city proper can be derived from the agricultural carrying capacity of Sagalassos’ primary catchment area of the Ağlasun and Yeşilbaşköy valleys (some 29 km²). Although various methods exist, assuming amount of land needed per family of 5 [35, 36], or the agricultural yield and calorific need per person per year [37, 38], all indicate a sustained population of 2,000-4,500 people, which does not include the influx of agricultural products from the wider territory of Sagalassos, which measured c. 1,200 km².

Apart from urban inhabitants, many people lived in the territory of Sagalassos, working as farmers, overseers etc. The ancient author Livy (38.15 – late 1st century BC-early 1st century AD) noted that the region of Sagalassos was rich in all kinds of fruit and in his Novella (XXIV.1, c. 535-536 AD) emperor Justinian refers to the populous villages of Pisidia. To reconstruct the population of the countryside, scholars have either relied upon urbanisation ratios between the population of the urban centre and the countryside [29, 39]. Recent scholarship seems to suggest that these ratios are not uniform for Antiquity, but rather dependent on the social and economic frameworks of a particular period. For example, in Classical Greek cities, a high urbanisation ratio is assumed (varying 80-66%), whereby the most people, including farmers tilling the countryside, are living in cities. For the Roman Republican and Imperial periods a much lower urbanisation rate of c.10-25% is suggested on the basis of city sizes and the number of sites in the countryside [32, 40]. This suggests that by the Roman period, many more people were living in smaller rural settlements outside the cities.

For Sagalassos, using the broader range of 1,500-5,000 inhabitants, this results in a total population (urban+rural) of 6,000-50,000. In terms of population density, this means a density of c. 5-42 persons/km². In comparison to published ancient population density reconstructions, this is relatively low with, for instance, Roman Italy's population density calculated at c. 20-90 pers./km², which was highly urbanized during the Republican and the Empire [40]. Yet in comparison to estimates for Roman Anatolia at 14 pers./km² the reconstructed population densities hold up well [41, 42]. Considering the latter estimate included vast areas of Anatolia, which were very sparsely settled in Roman times, such as Cappadocia, Galatia and Pontus, where cities often have territories of many thousands of square kilometers, a higher density of up to 42 pers./km² fits well with the more densely settled region of South-Western Turkey, where territories range from several hundred to two thousand square kilometers [27]. Therefore, a total population in the territory of Sagalassos in the tens of thousands of inhabitants is very likely.

**References**

[1] Leonard, J.A., Shanks, O., Hofreiter, M., Kreuz, E., Hodges, L., Ream, W., Wayne, R.K. & Fleischer, R.C. 2007 Animal DNA in PCR reagents plagues ancient DNA research. *JAS* **34**, 1361-1366. (doi:<http://dx.doi.org/10.1016/j.jas.2006.10.023)>.

[2] Champlot, S., Berthelot, C., Pruvost, M., Bennett, E.A., Grange, T. & Geigl, E.M. 2010 An efficient multistrategy DNA decontamination procedure of PCR reagents for hypersensitive PCR applications. *PLoS One* **5**. (doi:10.1371/journal.pone.0013042).

[3] Pruvost, M., Grange, T. & Geigl, E.-M. 2005 Minimizing DNA-contamination by using UNG-coupled quantitative real-time PCR (UQPCR) on degraded DNA samples: application to ancient DNA studies. *Biotechniques* **38**, 569-575.

[4] Ottoni, C., Ricaut, F.X., Vanderheyden, N., Brucato, N., Waelkens, M. & Decorte, R. 2011 Mitochondrial analysis of a Byzantine population reveals the differential impact of multiple historical events in South Anatolia. *Eur. J. Hum. Genet.* **19**, 571-576. (doi:10.1038/ejhg.2010.230).

[5] Ottoni, C., Girdland Flink, L., Evin, A., Georg, C., De Cupere, B., Van Neer, W., Bartosiewicz, L., Linderholm, A., Barnett, R., Peters, J., et al. 2013 Pig Domestication and Human-Mediated Dispersal in Western Eurasia Revealed through Ancient DNA and Geometric Morphometrics. *Mol. Biol. Evol.* (doi:mss261 [pii]10.1093/molbev/mss261 [doi]).

[6] Arndt, A., Van Neer, W., Hellemans, B., Robben, J., Volckaert, F. & Waelkens, M. 2003 Roman trade relationships at Sagalassos (Turkey) elucidated by ancient DNA of fish remains. *JAS* **30**, 1095-1105.

[7] Vermoere, M., Degryse, P., Vanhecke, L., Muchez, P., Paulissen, E., Smets, E. & Waelkens, M. 1999 Pollen analysis of two travertine sections in Basköy (southwestern Turkey): implications for environmental conditions during the early Holocene. *Review of Palaeobotany and Palynology* **105**, 93-110.

[8] Allentoft, M.E., Collins, M., Harker, D., Haile, J., Oskam, C.L., Hale, M.L., Campos, P.F., Samaniego, J.A., Gilbert, M.T., Willerslev, E., et al. 2012 The half-life of DNA in bone: measuring decay kinetics in 158 dated fossils. *Proc. Biol. Sci.* **279**, 4724-4733. (doi:10.1098/rspb.2012.1745).

[9] van Oven, M. & Kayser, M. 2009 Updated comprehensive phylogenetic tree of global human mitochondrial DNA variation. *Hum. Mutat.* **30**, E386-E394.

[10] Jehaes, E. 1998 Optimization of Methods and Procedures for the Analysis of mtDNA Sequences and their Applications in Molecular Archaeological and Historical Finds. Leuven, Leuven University Press.

[11] Excoffier, L. & Lischer, H.E.L. 2010 Arlequin suite ver 3.5: a new series of programs to perform population genetics analyses under Linux and Windows. *Mol. Ecol. Resour.* **10**, 564-567. (doi:10.1111/j.1755-0998.2010.02847.x).

[12] Benjamini, Y. & Hochberg, Y. 1995 Controlling the False Discovery Rate: A Practical and Powerful Approach to Multiple Testing. *Journal of the Royal Statistical Society. Series B (Methodological)* **57**, 289-300. (doi:10.2307/2346101).

[13] Team, R.D.C. 2008 R: A language and environment for statistical computing. . (Vienna (Austria), R Foundation for Statistical Computing.

[14] Bandelt, H.J., Forster, P. & Rohl, A. 1999 Median-joining networks for inferring intraspecific phylogenies. *Mol. Biol. Evol.* **16**, 37-48.

[15] Allen, P. 1979 The Justinian Plague. *Byzantion* **49**, 5-20.

[16] Anderson, C.N.K., Ramakrishnan, U., Chan, Y.L. & Hadly, E.A. 2005 Serial SimCoal: A population genetics model for data from multiple populations and points in time. *Bioinformatics* **21**, 1733-1734. (doi:10.1093/bioinformatics/bti154).

[17] Excoffier, L., Novembre, J. & Schneider, S. 2000 SIMCOAL: a general coalescent program for the simulation of molecular data in interconnected populations with arbitrary demography. *J. Hered.* **91**, 506-509.

[18] Endicott, P. & Ho, S.Y.W. 2008 A Bayesian Evaluation of Human Mitochondrial Substitution Rates. *The American Journal of Human Genetics* **82**, 895-902. (doi:10.1016/j.ajhg.2008.01.019).

[19] Kimura, M. 1980 A simple method for estimating evolutionary rates of base substitutions through comparative studies of nucleotide sequences. *J. Mol. Evol.*, 111-120.

[20] Fenner, J.N. 2005 Cross-cultural estimation of the human generation interval for use in genetics-based population divergence studies. *Am. J. Phys. Anthropol.* **128**, 415-423. (doi:10.1002/ajpa.20188).

[21] Beaumont, M. 2008 Joint determination of topology, divergence time and immigration in population trees. In *Simulation, Genetics and Human Prehistory* (eds. S. Matsumura, P. Forster & C. Renfrew), pp. 135-154. Cambridge (UK), McDonald Institute for Archaeological Research.

[22] Beaumont, M.A., Zhang, W. & Balding, D.J. 2002 Approximate Bayesian computation in population genetics. *Genetics* **162**, 2025-2035.

[23] Csilléry, K., François, O. & Blum, M.G.B. 2012 abc: an R package for approximate Bayesian computation (ABC). *Methods Ecol. Evol.* **3**, 475-479. (doi:10.1111/j.2041-210X.2011.00179.x).

[24] Rasteiro, R. & Chikhi, L. 2013 Female and Male Perspectives on the Neolithic Transition in Europe: Clues from Ancient and Modern Genetic Data. *PLoS One* **8**, e60944. (doi:10.1371/journal.pone.0060944).

[25] Hudson, R.R. 1990 Gene genealogies and the coalescent process. In *Oxford surveys in Evolutionary Biology* (eds. D. Futuyma & J. Antonovics), pp. 1-44. New York, Oxford University Press.

[26] Hansen, M.H. 2006 *The Shotgun Method. The Demography of the Ancient Greek City-State Culture*. Columbia & London, University of Missouri Press.

[27] Bowman, A. & Wilson, A. 2011 *Settlement, Urbanization, and Population*. Oxford, Oxford University Press.

[28] Willet, R. 2012 Whirlwind of numbers - demographic experiments for Roman Corinth. *Ancient Society* **42**, 127-158.

[29] Bintliff, J.L. 1997 Further considerations on the population of ancient Boeotia. In *Recent Developments in the History and Archaeology of Central Greece: Proceedings of the 6th International Boeotian Conference* (ed. J.L. Bintliff), pp. 231-252. Oxford, Archaeopress.

[30] Price, S. 2011 Estimating Ancient Greek Populations. The Evidence of Field Survey. In *Settlement, Urbanization, and Population* (eds. A. Bowman & A. Wilson), pp. 17-35. Oxford, Oxford University Press.

[31] Bagnall, R.S. & Frier, B.W. 1994 *The demography of Roman Egypt*. Cambridge, Cambridge University Press.

[32] Ligt, L.d. 2012 *Peasants, Citizens and Soldiers. Studies in the Demographic History of Roman Italy 225 BC - AD 100*. Cambridge, Cambridge University Press.

[33] Fentress, E. & Badel, J.P. 2003 Cosa in the Republic and early Empire. In *Cosa V. An intermittent town, Excavations 1991-1997* (ed. E. Fentress), pp. 13-62. Ann Arbor, University of Michigan Press.

[34] Zanker, P. 1998 *Pompeii. Public and Private Life*. Cambridge (MA) & London, Harvard University Press.

[35] Angelis, F.D. 2000 Estimating the Agricultural Base of Greek Sicily. *PBSR* **68**, 111-148.

[36] Bintliff, J.L. 2002 Rethinking early Mediterranean urbanism. In *Festschrift Für Manfred Korfmann, Mauerschau* (pp. 153-177. Tübingen, Verlag Bernhard Albert Greiner.

[37] Engels, D.W. 1990 *Roman Corinth: an Alternative Model for the Classical City*. Chicago, University of Chicago Press.

[38] Garnsey, P. 2004 *Cities, Peasants and Food in Classical Antiquity*. Cambridge, Cambridge University Press.

[39] Hansen, M.H. 2004 The Concept of the Consumption City Applied to the Greek Polis. In *Once Again: Studies in the Ancient Greek Polis* (ed. T.H. Nielsen), pp. 9-47. Stuttgart, Franz Steiner Verlag.

[40] Graaf, P.d. 2012 *Late Republican-early Imperial Regional Italian Landscapes and Demography*. Oxford, Archaeopress.

[41] Scheidel, W. 2007 Demography. In *The Cambridge economic history of the Greco-Roman world* (eds. W. Scheidel, I. Morris & R. Saller), pp. 38-86. Cambridge, Cambridge University Press.

[42] Frier, B.W. 2000 Demography. In *The Cambridge Ancient History (2nd edition) Volume XI The High Empire, A.D. 70–192* (eds. A. Bowman, P. Garnsey & D. Rathbone), pp. 113-143. Cambridge, Cambridge University Press.
